# Sublethal Levels of Antibiotics Promote Bacterial Persistence in Epithelial Cells

**DOI:** 10.1002/advs.201900840

**Published:** 2020-07-27

**Authors:** Xiaoye Liu, Fei Liu, Shuangyang Ding, Jianzhong Shen, Kui Zhu

**Affiliations:** ^1^ Beijing Advanced Innovation Center for Food Nutrition and Human Health College of Veterinary Medicine China Agricultural University No. 2 Yuanmingyuan West Road Beijing 100193 China; ^2^ National Center for Veterinary Drug Safety Evaluation College of Veterinary Medicine China Agricultural University Beijing 100193 China; ^3^ Beijing Key Laboratory of Detection Technology for Animal‐Derived Food Safety and Beijing Laboratory for Food Quality and Safety China Agricultural University Beijing 100193 China

**Keywords:** antibiotic, antibiotic tolerance, autophagy, bacteria, epithelial cells

## Abstract

Antibiotic therapy and host cells frequently fail to eliminate invasive bacterial pathogens due to the emergence of antibiotic resistance, resulting in the relapse and recurrence of infections. Bacteria evolve various strategies to persist and survive in epithelial cells, a front‐line barrier of host tissues counteracting invasion; however, it remains unclear how bacteria hijack cellular responses to promote cytoplasmic survival under antibiotic therapy. Here, it is demonstrated that extracellular bacteria show invasive behavior and survive in epithelial cells in both in vivo and in vitro models, to increase antibiotic tolerance. In turn, sublethal levels of antibiotics increase bacterial invasion through promoting the production of bacterial virulence factors. Furthermore, antibiotic treatments interrupt lysosomal acidification in autophagy due to the internalized bacteria, using *Bacillus cereus* and ciprofloxacin as a model. In addition, it is found that sublethal levels of ciprofloxacin cause mitochondrial dysfunction and reactive oxygen species (ROS) accumulation to impair lysosomal vascular tape ATPase (V‐ATPase) to further promote bacterial persistence. Collectively, these results highlight the potential of host cells mediated antibiotic tolerance, which markedly compromises antibiotic efficacy and worsens the outcomes of infection.

## Graphical abstract

Many extracellular bacteria are shown to be able to invade and survive in epithelial cells in both in vivo and in vitro models. Such adaption endows bacteria with common tolerance to multiple antibiotics. Sublethal levels of antibiotics not only promote the production of bacterial toxins to increase bacterial invasion, but also cause mitochondrial dysfunction and retard the clearance of internalized bacteria.

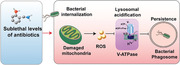

## 1 Introduction

The escalating crisis of antibiotic resistance calls for new antibiotics and strategies to combat bacterial pathogens associated infections.^[^ ^1^ ^]^ Discovering new antibiotics is challenging nowadays,^[^ ^2^ ^]^ it is therefore crucial that alternative solutions are urgently required to address this problem. One approach is to develop ways to revitalize existing antibiotics,^[^ ^3^, ^4^, ^5^ ^]^ to kill/inhibit multidrug resistant pathogens. To achieve such goal, we need further mechanistic understandings of the diverse ways by which bacteria survive under antibiotic therapy. Indeed, the relapse and recurrence of infections after treatments suggest that many failures of antibiotic therapy are caused by antibiotic tolerance of bacterial pathogens.^[^ ^6^, ^7^ ^]^ Unlike antibiotic resistant bacteria which inherit or acquire mutations,^[^ ^8^ ^]^ antibiotic tolerance is the capability of individual bacteria or bacterial populations to survive antibiotic stresses without genetic changes.^[^ ^9^, ^10^ ^]^ Phenotypic tolerance to antibiotics in bacteria with a transient, dormant, or non‐dividing status is usually induced by intermittent antibiotic exposures,^[^ ^11^, ^12^ ^]^ starvation,^[^ ^13^, ^14^ ^]^ or host environment.^[^ ^15^ ^]^Antibiotic tolerance can facilitate the evolution of antibiotic resistance.^[^ ^16^, ^17^ ^]^ For instance, *Salmonella* Typhimurium forms persisters to promote the dissemination of antibiotic resistance plasmids.^[^ ^18^ ^]^ However, it remains largely unclear what the driving force for the emergence of antibiotic tolerance is, particularly in vivo.

Upon infections particularly persistent infections,^[^ ^19^, ^20^ ^]^ sophisticated defense responses are sequentially activated in hosts to clear bacterial invaders,^[^ ^21^, ^22^, ^23^ ^]^ together with other therapeutic strategies including antibiotic therapy. Epithelial cells consist of a front‐line barrier counteracting such invasion in hosts.^[^ ^24^, ^25^ ^]^ Epithelial cells play a crucial role in bridging the interactions between bacteria and host responses,^[^ ^26^, ^27^ ^]^ which may determine the efficacy of antibiotics and even the outcomes as well.^[^ ^28^ ^]^ Epithelial cells usually harness multiple defensive mechanisms against bacterial invasion including cell integrity, rapid cell turnover, apoptosis, and autophagy.^[^ ^29^, ^30^ ^]^ On the other hand, many bacteria evolve adaptive strategies to circumvent the clearance by modulating cellular signals. Compared to obligate and facultative intracellular bacterial pathogens such as *Mycobacterium tuberculosis* and *S*. Typhimurium,^[^ ^31^ ^]^ many extracellular bacterial pathogens such as *Staphylococcus aureus*, are able to invade, survive, and persist in the cytosol of epithelial cells.^[^ ^32^ ^]^ Once such bacteria survive in host cells, they act as “Trojan horses” to tolerate various stresses including antibiotic therapy. For example, survival of *S. aureus* within cells increases its ability against the treatment of hundreds of folds of vancomycin.^[^ ^33^ ^]^ Meanwhile, other extracellular bacteria such as *Bacillus cereus*, *Escherichia coli*, *Enterococcus faecalis*, and *Vibrio parahaemolyticus* have also been shown to replicate in diverse cells.^[^ ^34^, ^35^, ^36^, ^37^ ^]^ Therefore, we hypothesized that survival of extracellular bacteria in epithelial cells served as a reservoir to elude antibiotic treatments. The persistence of bacteria in the cytosol may enable the emergence of antibiotic tolerance to cause the recurrent infections.

In this study, we first observed that eight species of extracellular bacteria could invade epithelial cells in both in vitro and in vivo models. Sublethal levels of antibiotics promoted the production of virulence factors to enhance bacterial invasion. Then, we found the low level of ciprofloxacin caused mitochondrial dysfunction and ROS accumulation by inhibiting lysosomal V‐ATPase to further promote *B. cereus* persistence. Lastly, the internalized bacteria (including *E. coli* and *B. cereus*) survived in the cytoplasm by paralyzing the acidification of autophagosomes.

## 2 Results

### 2.1 Epithelial Cells Protect Internalized Bacteria from Antibiotic Treatments

**Figure 1:**
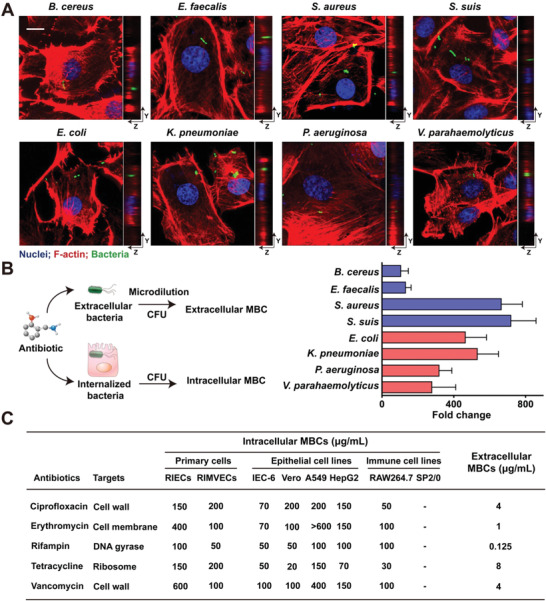
Increased tolerance of bacteria in epithelial cells against antibiotics. A) Bacterial internalization in epithelial cells. IEC‐6 cells were infected with diverse bacteria for 2 h, including *B. cereus* NVH0075/95, *E. coli* ATCC25922, *E. faecalis* ATCC29212, *S. aureus* ATCC29213, *S. suis* CQ2B50, *K. pneumoniae* 1202, *P. aeruginosa* PAO1, and *V. parahaemolyticus* ATCC17802. F‐actin were stained by rhodamine phalloidin (red) and nuclei were counterstained with DAPI (blue). *B. cereus* expressing GFP (green) and the other bacteria were labeled with pHrodo (green). Scale bar = 10 µm. B) Internalized bacteria were tolerant to antibiotics. Experimental workflow of intracellular MBC and extracellular MBC assays (left). Fold changes were calculated as the ratios of the values of intracellular MBC to values of extracellular MBC (right). Ciprofloxacin was used for all bacteria tested, except *S. suis* with ampicillin and *K. pneumoniae* with polymyxin B. C) The intracellular and extracellular MBCs. Extracellular MBCs were the minimum antibiotic doses that prevented the survival of *B. cereus* NVH0075/95 (with > 99.9% bacteria dead), which was detected based on the extracellular MICs that prevented bacterial growth in DMEM. Intracellular MBCs were the minimum doses that prevented the survival of internalized *B. cereus* NVH0075/95 in various mammalian cells (> denoted the continuous growth of bacteria under the maximum dose of antibiotics tested). Data were presented as means from three different experiments.

To get better understanding of post‐antibiotic expansion of bacterial pathogens in clinic,^[^ ^28^, ^38^, ^39^ ^]^ we employed a mouse model orally infected with *B. cereus* and *E. coli*. We observed the presence of *B. cereus* and *E. coli* in the epithelial cells, particularly in the ileum (Figure S1A,B, Supporting Information). Then we prepared primary rat intestinal epithelial cells (RIECs) and infected with each of multiple pathogens, including four Gram‐positive bacteria *Bacillus cereus*, *Enterococcus faecalis*, *Staphylococcus aureus*, and *Streptococcus suis*, and four Gram‐negative bacteria *Escherichia coli*, *Klebsiella pneumoniae*, *Pseudomonas aeruginosa*, and *V. parahaemolyticus*. Consistently, we found that all bacteria could invade RIECs (Figure S1C, Supporting Information) and similar phenomenon was observed in diverse epithelial cell lines including rat small intestine cell (IEC‐6) cells (**Figure** **1**A), lung carcinoma cell (A549), human hepatocellular carcinoma (HepG2), and African green monkey kidney cell (Vero cells) (Figure S1D–F, Supporting Information). Unlike macrophages such as RAW 264.7 cells which actively phagocytose bacteria, non‐phagocytic immune cells such as mouse hybridoma cells (S/P20 cells) could not be infected (Figure S1G, Supporting Information). These findings suggested that extracellular bacteria could invade epithelial cells in both in vivo and in vitro models.

To compare the efficacy of antibiotics against bacteria in the presence and absence of cells, we used IEC‐6 cells as a model. Compared to the extracellular minimum bactericidal concentrations (MBCs) of eight bacteria, we observed a significant increase of intracellular MBCs with 107 to 667‐fold increases (Figure 1B). It indicated that antibiotic efficacy dramatically decreased against internalized bacteria, although these bacteria were susceptible to antibiotics in the absence of cells. Subsequently, we tested the intracellular MBCs of multiple antibiotics with different modes of action against *B. cereus*. It showed that the values of intracellular MBCs significantly increased in the presence of diverse cells (Figure 1C), suggesting a common mechanism of antibiotic tolerance. Additionally, we extended to examine the antibacterial activity against *E. coli*, *S. aureus*, and *V. parahaemolyticus* in different types of epithelial cells. Consistent with the observation in *B. cereus*, we found dramatically increased antibiotic tolerance (Table S5, Supporting Information). Altogether, these results suggested that epithelial cells could protect internalized bacteria from high levels of antibiotic treatments.

### 2.2 Sublethal Levels of Antibiotics Facilitate Bacterial Invasion to Epithelial Cells

**Figure 2:**
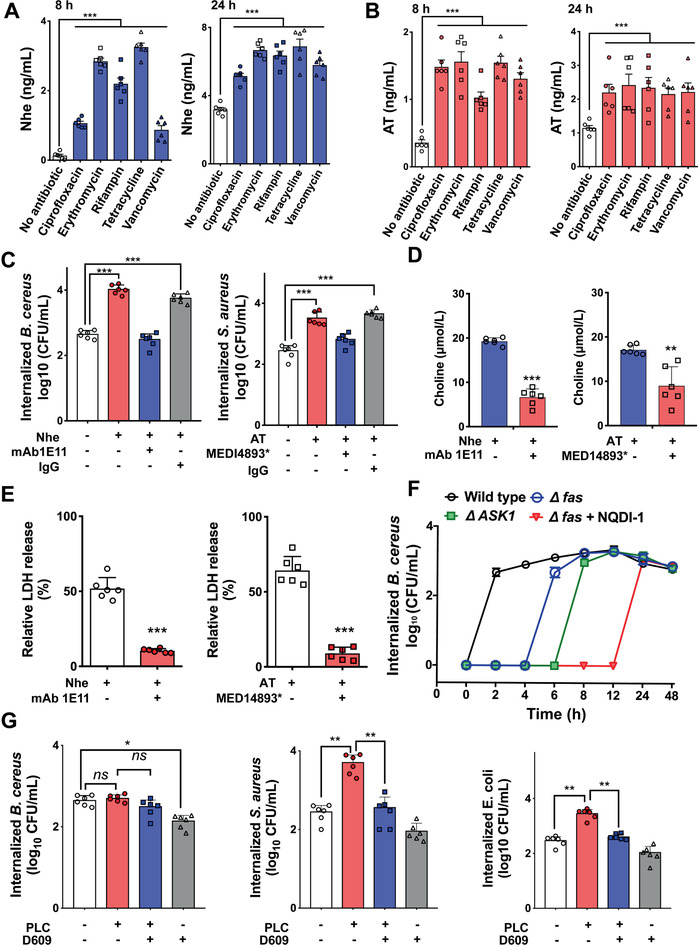
Sublethal levels of antibiotics promote toxin production facilitating bacterial invasion. A,B) Antibiotics promoted the production of bacterial toxins. IEC‐6 cells were infected with *B. cereus* NVH0075/95 or *S. aureus* ATCC29213 at the MOI of 40 in the presence of antibiotics (0.5 µg mL^−1^ ciprofloxacin, 0.25 µg mL^−1^ erythromycin, 4 µg mL^−1^ tetracycline, 0.625 µg mL^−1^ rifampin, and 2 µg mL^−1^ vancomycin) for 8 h. The levels of Nhe from *B. cereus* or AT (*α*‐toxin) from *S. aureus* were measured using enzyme immunoassays. C) Exogenous addition of Nhe and AT promoted the invasion of *B. cereus* NVH0075/95 and *S. aureus* ATCC29213, respectively. Antibodies mAb 1E11 neutralizing Nhe, MEDI4893* neutralizing AT and rabbit anti‐IgG antibody were used. D,E) Cytotoxicity of bacterial toxins. Accelerated release of choline (D) and LDH (E) from IEC‐6 cells treated with Nhe (23 ng mL^−1^) or AT (ng mL^−1^) and their corresponding neutralizing antibodies (anti‐NheB mAb 1E11, 2 µg mL^−1^, and anti‐*α*‐toxin mAb, MEDI4893*, 5 µg mL^−1^) for 2 h. F) Fas and ASK1 pathways induced were involved against *B. cereus* invasion. Numbers of internalized bacteria in the wide‐type and mutant (*Δfas* or *ΔASK1*) Vero cells infected with *B. cereus* NVH0075/95 (MOI = 40) in 48 h. NQDI1 is a specific inhibitor of ASK1 and results are representative of three independent assays. G) Additional PLC increased bacterial invasion. PLC is derived from *B. cereus* and D609 is a specific inhibitor of PLC. Data are represented as mean ± SEM, *p < 0.05, **p < 0.01, ***p < 0.001, *n* = 6.

To dissect epithelial cells mediated antibiotic tolerance, we first focused on the effect of antibiotics on bacterial invasion, which is the restricted step to form the cell‐mediated tolerance. It is well known that bacterial toxins can facilitate bacteria entry into host cells.^[^ ^40^, ^41^, ^42^, ^43^, ^44^ ^]^ Therefore, we measured the production of two major toxins in *B. cereus* (non‐hemolytic enterotoxin, Nhe) and *S. aureus* (*α*‐toxin, AT) in the presence of sublethal levels of antibiotics. Results showed that such antibiotics promoted toxin productions in a time dependent manner (**Figure** **2**A,B). In turn, Nhe and AT facilitated the invasion of *B. cereus* and *S. aureus*, consistent with the notion that bacterial toxins are crucial to accelerate invasion.^[^ ^40^, ^41^, ^44^ ^]^ Correspondingly, addition of neutralizing antibodies mAb 1E11 or MED14893* could abolish the potentiated invasion (Figure 2C). Furthermore, we found that these bacterial toxins damaged the integrity of cell membrane, as indicated by the release of choline and lactate dehydrogenase (LDH) (Figure 2D,E). Lastly, we deciphered the involved signal pathways during invasion. According to our previous findings that both factor associated suicide (Fas) and signal‐regulating kinase (ASK1) are important to trigger apoptosis in *B. cereus* associated infections,^[^ ^45^ ^]^ we therefore quantified bacterial numbers in mutant cells with the deletion of either *fas* or *ASK1* genes. Interestingly, the invasion of *B. cereus* dramatically postponed in the mutants, particularly when both signals were simultaneously inhibited (Figure 2F). It indicated that *B. cereus* could hijack cellular responses to coordinate invasion. Additionally, we evaluated the role of other bacterial virulence factors such as phospholipase in bacterial invasion. Exogenous addition of phospholipase C (PLC) derived from *B. cereus* could promote the invasion for either *S. aureus* or *E. coli* (Figure 2G). Although the invasion of *B. cereus* was not enhanced by additional PLC, its specific inhibitor (D609) reduced the invasion of *B. cereus*. Taken together, these data suggested that bacteria could harness versatile virulence factors to achieve the invasion of epithelial cells.

**Figure 3:**
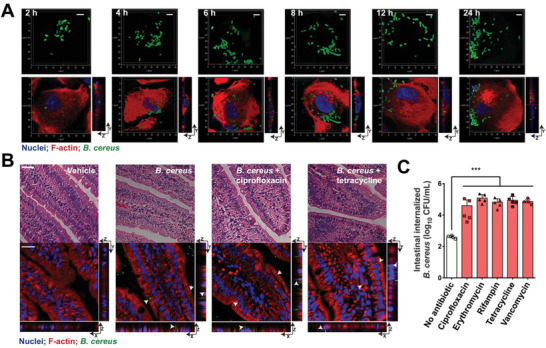
Sublethal levels of antibiotics promote bacterial internalization in vivo. A) Growth dynamics of internalized *B. cereus* in IEC‐6 cells. Confocal images of IEC‐6 cells infected with GFP‐labeled was counterstained *B. cereus* (green, MOI = 40) for 2 to 24 h. The 3D images were obtained by CLSM, F‐actin was visualized using rhodamine phalloidin (red) and nuclei with DAPI (blue). Scale bars: 5 µm. B) Antibiotics promoted bacterial internalization in mice. Mice were infected with 1 × 10^9^ CFUs of *B. cereus* under the treatments of 0.5‐fold extracellular MICs of each antibiotic for 24 h. *B. cereus* were shown in green (arrowheads). Sections of ileum were stained by hematoxylin–eosin (H&E) stain (upper) and confocal images (bottom). Scar bars = 20 µm. C) Antibiotics promoted bacterial internalization in the small intestine of mice. The numbers of *B. cereus* in ileums were counted in the presence of each antibiotic at the levels of 0.5 extracellular MICs. ***p < 0.0001, *n* = 5 mice per group.

Next, we investigated how antibiotics modulated the intracellular lifestyle of invasive bacteria. First, we observed that the proliferation of persistent *B. cereus* in epithelial cells in a time depend manner (**Figure** **3**A). To quantify the efficacy of bacteria invading epithelial cells, we found that the increased multiplicity of infection (MOI, the number of bacteria that are added per cell during infection) and long infection time resulting in more internalized bacteria, survival and replication in cells (Figure S2, Supporting Information). Meanwhile, we observed that sublethal levels of antibiotics had no effect on the growth of IEC‐6 cells (Figure S3A, Supporting Information). Then we quantified the concentrations of antibiotics in the cytosol of IEC‐6 cells infected with *B. cereus* based on liquid chromatography‐tandem mass analysis (LC‐MS/MS) (Figure S3B and Table S2, Supporting Information). Compared to the abundant extracellular antibiotics, accumulated antibiotics in cells consisted only about 0.28–14.34% of the total (Figure S3C, Supporting Information). Given that sufficient levels of antibiotics are prerequisite to inhibit bacterial growth,^[^ ^13^, ^14^, ^17^ ^]^ such low levels of antibiotics in the cytosol could not reach sufficient concentrations to kill/inhibit bacteria. Thus, we used sublethal levels of antibiotics to treat infected mice and observed that such antibiotics could promote *B. cereus* internalization (Figure 3B,C). Moreover, antibiotic treatments promoted the internalization of *B. cereus* in a time dependent manner (Figure S4A, Supporting Information). In addition, long‐term exposures to sublethal levels of antibiotics except tetracycline advanced the survival of internalized *B. cereus* (Figure S4B–D, Supporting Information). It might be due to either the intrinsic property of tetracycline or that partial bacteria escaped from the epithelial cells treated with tetracycline.

Although *B. cereus* is a spore forming bacterium, we found that the vegetative cells comprised a large proportion of bacterial numbers in IEC‐6 cells (Figure S5A,B, Supporting Information), although the increased numbers of spores were observed as well. It is in agreement with that sub‐lethal levels of antibiotics promoted diverse non‐spore‐forming bacteria invading epithelial cells (Figures 1 and 2). Furthermore, the nutrients in the intracellular environment are limited for the survival of invaded bacteria.^[^ ^13^ ^]^ Compared to the abounding nutrients in media (Figure S5C, Supporting Information), we found the upregulation of starvation response‐related genes in both *B. cereus* (*yjbM* and *yawC*) and *E. coli* (*reclA* and *spoT*), under antibiotic treatments (Figure S5D, Supporting Information). It revealed that bacteria adapted to the conditions of deprived nutrients in epithelial cytoplasm. It is worth noting that epithelial cells always induce autophagy upon starvation.^[^ ^46^, ^47^ ^]^

### 2.3 Sublethal Levels of Antibiotics Promote Bacterial Persistence by Inducing Autophagy Arrest

**Figure 4:**
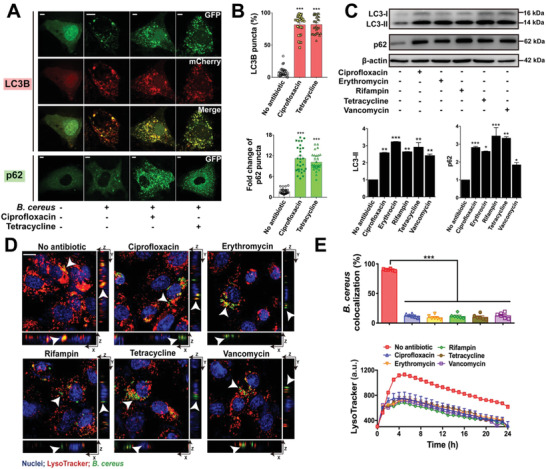
Antibiotics interrupt autophagy to assist bacterial survival. A) Ciprofloxacin and tetracycline suppressed autophagy. IEC‐6 cells were transfected with modified adenoviruses (Ad‐mcherry‐GFP‐LC3B and Ad‐GFP‐p62) and infected with *B. cereus* NVH0075/95 (MOI = 40) under antibiotic treatments (0.5 µg mL^−1^ ciprofloxacin and 4 µg mL^−1^ tetracycline) for 8 h. Merge of LC3B presented either non‐autophagy (yellow LC3B dispersion), autophagy (red LC3B puncta) or autophagy arrest (yellow LC3B puncta). Scar bars = 3 µm. B) Percentage of LC3B puncta and p62 puncta was quantified from (A). The percentage of LC3B was calculated from the ratio of yellow LC3B puncta to red LC3B puncta. The fold changes of p62 puncta were compared to the untreated group. Both LC3B and p62 puncta were randomly selected from 30 cells. C) Expression of p62 and LC3 in IEC‐6 cells infected with *B. cereus* NVH0075/95 (MOI = 40) under antibiotics. Both the expression of LC3‐II and p62 were normalized to the levels of *β*‐actin based on Western blot analysis. Data are showed as means ± SEM (*n* = 3, *p < 0.05, **p < 0.001, ***p < 0.0001). D) Antibiotic treatments decreased the colocalization of bacteria with acidified compartments. IEC‐6 cells were infected with GFP labeled *B. cereus* (MOI = 40) for 8 h, in the presence of each antibiotics of 0.5‐fold of extracellular MICs (0.5 µg mL^−1^ciprofloxacin, 0.25 µg mL^−1^ erythromycin, 4 µg mL^−1^ tetracycline, 0.625 µg mL^−1^ rifampin, and 2 µg mL^−1^ vancomycin). Cells were stained by Hoechst (nucli, blue) and LysoTracker (acidified compartments, red). The arrowheads mark the colocalization of bacteria with acidified compartments. E) Antibiotics decreased acidification of compartments. Quantified bacterial colocalization with acidified compartments from **D** was showed (upper). Dynamic curves of acidified compartments probing with LysoTracker (bottom). Data are represented as mean ± SEM (*n* = 6, ***p < 0.0001).

Host cells always initiate multiple strategies such as autophagy to defense invasive bacteria.^[^ ^47^, ^48^, ^49^ ^]^ To better understand bacterial survival in cells, we hypothesized that antibiotics promoted bacteria persistence in the cytosol through hijacking autophagy. We dissected the complicated signaling cascade of autophagy using two markers (light chain 3, LC3; p62/sequestosome‐1, p62/SQSTM1).^[^ ^50^ ^]^ We first found that *B. cereus* interrupted the process of autophagy in a time dependent manner by regulating the expression of LC3‐II and p62 (Figure S6, Supporting Information). Meanwhile, antibiotic treatments aggravated autophagy arrest with the increase of dual florescence labeled LC3 (**Figure** **4**A), while GFP labeled LC3 was quenched its GFP florescence in the acidic environment due to the fusion of autophagosome and lysosome.^[^ ^51^, ^52^ ^]^ Furthermore, antibiotics induced the increase of numbers of p62 puncta (Figure 4B), denoting the interruption of lysosomal degradation.^[^ ^30^, ^48^, ^51^ ^]^ In addition, antibiotics upregulated the expression of LC3‐II and p62 in IEC‐6 cells infected with *B. cereus* based on Western blot analysis (Figure 4C). These results showed that antibiotics facilitated the survival of *B. cereus* through suppressing autophagy. Similarly, we observed fluorescent patterns and increased expression of LC3 and p62 in IEC‐6 cells infected with *E. coli* (Figure S7, Supporting Information).

We next sought to elucidate the autophagy arrest of infected cells, using the inducer rapamycin and inhibitor chloroquine of autophagy. Antibiotics mediated autophagy arrest was rescued by rapamycin while enhanced by chloroquine (Figure S8A–C, Supporting Information). It suggested that antibiotics promoted bacteria inducing autophagy arrest. Internalized bacteria are usually delivered to the lysosomes for further degradation. The acidic microenvironment plays a critical role in maintaining the activity of lysosome.^[^ ^53^ ^]^ Thus, we tracked the acidic lysosomes in IEC‐6 cells and observed the decreased numbers of co‐localized *B. cereus* with acidic organelles in the presence of antibiotics (Figure 4D,E). To further characterize the reduced acidic organelles, we found that the labeled lysosomal membrane protein (LAMP1) was not colocalized with bacteria under antibiotic treatment (Figure S8D, Supporting Information). These results suggested that the process of acidification was impeded, to interrupt the degradation of engulfed bacteria.

### 2.4 Sublethal Level of Ciprofloxacin Cause Mitochondrial Dysfunction and Inhibition of Lysosomal V‐ATPase

**Figure 5:**
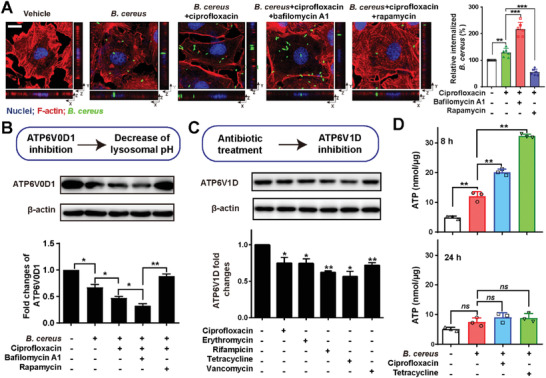
Sublethal levels antibiotics inhibited lysosomal V‐ATPase to facilitate bacteria survival. A) Inhibition of acidified lysosomes enhancing the intracellular survival of *B. cereus*. Bafilomycin A1 (100 nm) was used to inhibited V‐ATPase in IEC‐6 cells for 1 h. Rapamycin (100 nm) was employed to inhibit autophagy. The numbers of internalized bacteria were counted by the CFU assay. Results are shown as means ± SEM (*n* = 6, **p < 0.01, ***p < 0.001). Scale bar: 20 µm. B) Expression of ATP6V1D in lysosomes using Western blot. IEC‐6 cells were infected with *B. cereus* NVH0075/95 (MOI = 40) under ciprofloxacin treatment (0.5 µg mL^−1^). All proteins were normalized to the levels of *β*‐actin (compared to sole bacterial infectious group). Results are shown as means ± SEM (*p < 0.05; **p < 0.001). C) Increase of ATP levels under antibiotic exposure. IEC‐6 cells were infected with *B. cereus* under antibiotic treatments (0.5 µg mL^−1^ciprofloxacin, 0.25 µg mL^−1^ erythromycin, 4 µg mL^−1^ tetracycline, 0.625 µg mL^−1^ rifampin, and 2 µg mL^−1^ vancomycin). The release of ATP in the supernatants were measured by normalizing the ATP levels to the amount of proteins. Data are shown as mean ± SEM for at least three replicates (**p < 0.01). D) Expression of ATP6V0D in the lysosome of IEC‐6 cells. Cells were infected with *B. cereus* NVH0075/95 (MOI = 40) with the treatment of 0.5 µg mL^−1^ ciprofloxacin. Cells were pre‐incubated with bafilomycin A1 (100 × 10^−9^ m) for 1 h to inhibit V‐ATPase. Rapamycin (100 × 10^−9^ m) targeting mTOR was used as an inducer of autophagy. All proteins were normalized to the levels of *β*‐actin. Data are showed as means ± SEM (*p < 0.05, **p < 0.001, *n* = 3).

To further characterize how antibiotics impair lysosomal acidification, we focused on the lysosomal V‐ATPase complex, the workhorse for maintaining the acidic environment in lysosomes.^[^ ^54^ ^]^ We observed that antibiotic treatment promoted bacterial survival in the presence of specific inhibitor of V‐ATPase bafilomycin A1 using ciprofloxacin as an example (**Figure** **5**A; Figure S9A, Supporting Information). Bafilomycin A1 led to the inhibition of ATP6V0D1 (Figure 5B; Figure S9B, Supporting Information), a subunit of V0‐sector for H^+^ transporting. These findings are consistent with previous reports that *Streptococcus pyogenes* and *S*. Typhimurium modulate V‐ATPase to inhibit lysosomal acidification.^[^ ^55^, ^56^ ^]^ Activity of V‐ATPase plays a crucial role in the function of mammalian target of rapamycin (mTOR).^[^ ^57^ ^]^ We found that ciprofloxacin promoted the expression of the activated form of mTOR (phosphorylated mTOR, p‐mTOR) (Figure S8C, Supporting Information), confirming the inhibition of V‐ATPase.^[^ ^58^ ^]^ V‐ATPase particularly the V1‐sector, is responsible for ATP hydrolysis to maintain the pH gradient.^[^ ^59^ ^]^ Therefore, we tested the expression of ATP6V1D (a subunit of V1‐sector) and found the inhibited expression of ATP6V1D under ciprofloxacin treatment (Figure 5C; Figure S9C, Supporting Information). Correspondingly, the ATP level was significantly decreased under long‐term antibiotic treatments, whereas the compensatory ATP accumulation occurred at the early stage (Figure 5D).

**Figure 6:**
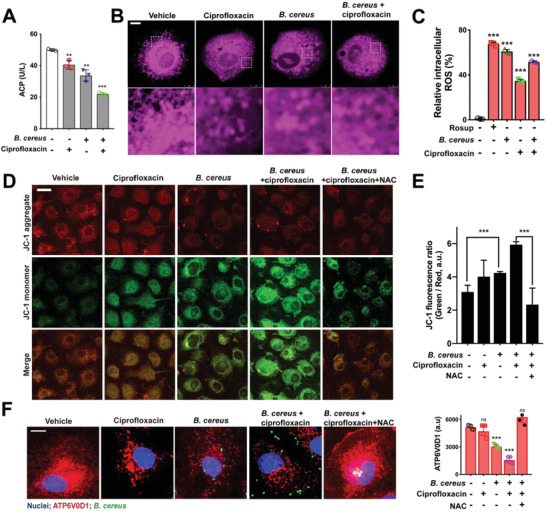
Antibiotics induced mitochondrial dysfunction causing inhibition of lysosomal V‐ATPase. A) Decreased activity of ACP. IEC‐6 cells were infected with *B. cereus* (MOI = 40) in the presence of sublethal level of 0.5 µg mL^−1^ ciprofloxacin for 8 h. Data are represented as mean ± SEM. (**p < 0.01, ***p < 0.001, *n* = 3). B) Damaged structure of mitochondria. The mitochondria in IEC‐6 cells treated with ciprofloxacin were tracked by mitotracker. C) Accumulation of intracellular ROS in IEC‐6 cells based on Flow cytometry analysis. Rosup (125 µg mL^−1^) was a positive control for the generation of ROS. Results are showed as mean ± SEM (***p < 0.001, *n* = 3). D) Changes of membrane potential (Δ*ψ*m) in mitochondria at 24 h infection. Confocal images of JC‐1 in IEC‐6 cells. JC‐1 is in red fluorescence when Δ*ψ*m is high, whereas in green when Δ*ψ*m is low. Scale bar: 25 µm. E) The radio of JC‐1 green to red fluorescence from (D). The radio of green fluorescence to red indicates that JC‐1 aggregates into monomers, representing the loss of Δ*ψ*m. NAC (5 mm) was used to eliminate intracellular ROS. F) Antibiotic treatment inhibited ATPV0D1. IEC‐6 cells infected with *B. cereus* were treated with sub‐lethal ciprofloxacin at concentrations of 0.5 µg mL^−1^. NAC is as a ROS scavenger. Expression of ATPV0D1 (red) was detected by immunofluorescence. Data are showed as mean ± SEM. (***p < 0.001, *ns*: *p* > 0.05, *n* = 3). Scale bar: 10 µm.

In addition, we found that inhibition of lysosomal acidification further decreased the activity of acid phosphatase (ACP) in the presence of ciprofloxacin (**Figure** **6**A). Decreased activity of ACP retards the clearance of damaged mitochondria,^[^ ^60^, ^61^ ^]^ leading to the inhibition of lysosomal acidification. Consistent with previous observations,^[^ ^62^, ^63^ ^]^ we found that ciprofloxacin caused morphological damage and dysfunction on mitochondria (Figure 6B), resulting in ROS accumulation (Figure 6C), with the decrease of membrane potential (Figure 6D,E). Exogenous addition of ROS scavenger NAC (*N*‐acetyl cysteine) could reverse the inhibition of ATP6V0D1 (Figure 6F). Taken together, these results demonstrated that sublethal levels of antibiotics induced mitochondrial dysfunction resulting in ROS accumulation, which may contribute to the inhibition of lysosomal acidification.^[^ ^64^, ^65^ ^]^

**Figure 7:**
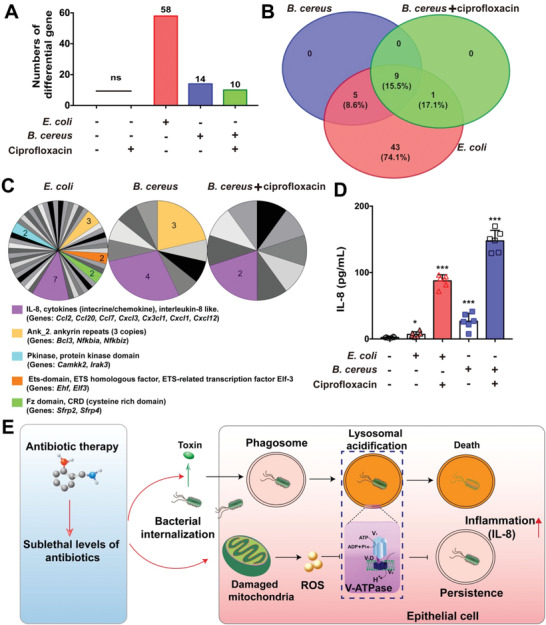
Transcriptome analysis of IEC‐6 infected with bacteria. A) Numbers of different genes in IEC‐6 cells infected with *E. coli* ATCC25922 or *B. cereus* NVH0075/95 in the presence and absence of 0.5 µg mL^−1^ ciprofloxacin at MOI of 40 for 8 h. Cellular RNA were sequenced by RNA‐Seq. The significant genes were calculated by comparing to the untreated cells, with DEseq2. Only genes with log_2_ fold Change≥2, FDR < 0.01 for further analysis. B) Venn diagram of (A). C) The families and domains of potential proteins. The numbers of related proteins were labeled and proteins were annotated using the Pfam database. D) Expression of IL‐8 in IEC‐6 cells. The cells were infected with either *B. cereus* or *E. coli* in the presence of 0.5 µg mL^−1^ ciprofloxacin. Data are presented as means ± SEM (*p < 0.05, ***p < 0.001, *n* = 3). E) Scheme of sublethal levels of antibiotics promote bacterial survival in host cells.

To further decipher the cellular responses, transcriptome analysis was employed to evaluate the gene expression of IEC‐6 cells infected with bacteria under ciprofloxacin treatment. Compared to the profile of cells infected with *E. coli*, there were fewer changes of gene expression in the cells infected with *B. cereus*, particularly in the presence of ciprofloxacin (**Figure** **7**A,B). Interestingly, we found that the upregulation of genes related to inflammatory cytokine interleukin‐8 (IL‐8) was presented in all treatments (Figure 7C). Then we quantified IL‐8 and confirmed that ciprofloxacin treatment enhanced the production of IL‐8 (Figure 7D). Although the regulation of IL‐8 remains unclear in the infected cells, implying that sublethal levels of antibiotics trigger a cascade of inflammation responses.^[^ ^66^ ^]^ Recently, the toxin Nhe of *B. cereus* has been demonstrated to activate the NLRP3 inflammasome.^[^ ^67^ ^]^ Thus, further works are needed to elucidate the inflammatory response pathway in host cells mediated antibiotic tolerance.

## 3 Discussion

Extracellular bacteria such as *B. cereus*, *E. coli*, and *S. aureus* usually cause systematic infections due to the translocation from the initially persisted sites.^[^ ^22^ ^]^ Failure of antibiotic therapy in clinic includes the emergence of antibiotic resistance and/or tolerance,^[^ ^3^, ^6^ ^]^ resulting in the disruption of normal microbiome particularly for gastrointestinal infections.^[^ ^15^ ^]^ Pathogenic bacteria subsequently invade the epithelium before the recovery of colonization resistance.^[^ ^68^ ^]^ Survival of bacteria in the cytosol of epithelial cells therefore obtains many benefits when extracellular bacteria manage to counteract the clearance of host. On one hand, such invasive bacteria are much easier to penetrate the first barrier for sequential dissemination from the infected sites. On the other, these bacteria acting as “Trojan horses” in epithelial cells achieve an economic way to sustain antibiotic stresses without genetic costs. In the present work, we found that many extracellular bacteria could invade and survive in diverse epithelial cells in both in vivo and in vitro models (Figure 1; Figure S1, Supporting Information), which is consistent with previous reports for *E. faecalis* and *V. parahaemolyticus*.^[^ ^29^, ^35^ ^]^ Consequently, epithelial cells provide special niches for these bacteria to tolerate multiple antibiotics, because antibiotics cannot accumulate to enough levels in the cytosol to kill/inhibit bacteria.^[^ ^69^ ^]^

The side effects of antibiotics on disrupting host microbiome have been well studied. For instance, antibiotic treatment depletes commensal butyrate‐producing *Clostridia* in the gut, leading to the increased epithelial oxygenation and expansion of *S*. Typhimurium.^[^ ^6^ ^]^ Furthermore, antibiotic‐associated diarrhea (AAD) is caused after antibiotic therapy, notoriously known for *Clostridium difficile* infection (CDI).^[^ ^70^ ^]^ However, the underlying mechanisms of post antibiotic expansion remain unclear. We here provide an alternative explanation that antibiotics promote colonization of extracellular bacteria in epithelial cells to cause secondary infections when the level of antibiotics decrease or fade away.

Host cells often eliminate the infected cells through apoptosis, autophagy, recruiting immune cells and other strategies. It has been shown that epithelial autophagy is essential for the defense against invasive bacterial pathogens.^[^ ^29^ ^]^ Surprisingly, we observed that the low levels of antibiotics could facilitate bacterial survival through autophagy arrest in epithelial cells (Figure 3; Figure S7, Supporting Information). Antibiotics blocked the fusion of autophagosome and lysosome by upregulating LC3 and p62 (Figure 3A,B). Similarly, *V. parahaemolyticus* and *Legionella pneumophila* modulate lysosomal acidification for survival.^[^ ^71^, ^72^ ^]^ We found that the inhibition of V‐ATPase impaired lysosomal acidification (Figure 4). In addition, both mitochondrial dysfunctions and damaged cell membrane contribute to ROS accumulation (Figure 4; Figure S9, Supporting Information), to further paralyze cellular homeostasis facilitating bacterial survival in the cytosol.

## 4 Conclusion

We describe a general observation that both Gram‐positive and Gram‐negative bacteria, known for their extracellular lifestyles, can invade and survive in diverse epithelial cells. Such adaption endows bacteria with the tolerance to multiple antibiotics (Figure 7E). Sublethal levels of antibiotics not only promote the production of bacterial toxins to increase invasion, but also cause mitochondrial dysfunction and ROS accumulation resulting in autophagy arrest. Our findings provide a framework for host cells mediated antibiotic tolerance in both in vivo and in vitro models, which will shed light on the better use of antibiotics and development of alternative strategies to either target the internalized bacteria or to boost the cellular defense of host cells, to reduce the recurrence of infections.

## 5 Experimental Section

##### Bacterial Strains and Mammalian Cells

Eight extracellular bacterial strains were used in this study (Table S1, Supporting Information). Four epithelial cell lines, two immune cell lines and two kinds of primary cell lines, were employed in this work (Table S2, Supporting Information). *Δfas* or *ΔASK1* cells were mutants of Vero cells constructed by CRISPR‐Cas9 knockout assay, according to the previously published method.^[^ ^45^ ^]^ Addition of NQDI1 (500 nm, Sigma‐Aldrich) in *Δfas* cells was to obtain the double knockdown of both Fas and ASK1 proteins on Vero cells, because NQDI1 was used as a specific inhibitor of ASK1.^[^ ^73^ ^]^ More details of the bacterial strains and mammalian cells used in this study are provided in Supporting Information.

##### Mammalian Cell and Mouse Infections

Mammalian cells were seeded at 1 × 10^5^ cells per well onto glass coverslips (14 mm, NEST) in 24‐well culture plates (Corning) to form monolayers. Then bacterial colonies were scraped and resuspended in PBS (0.01 m, pH = 7.2, Gibco) to pre‐incubate with pHrodo‐Green (Molecular Probes), except *B. cereus* pGFP 4412 expressing GFP. Finally, 4 × 10^6^ colony‐forming units (CFUs) bacteria were cocultured with mammalian cells.

5‐week‐old female ICR mice (*n* ≥ 5) were infected intragastrically with 200 µL bacteria (*B. cereus* NVH0075/95 and *E. coli* ATCC25922) in 0.9% saline solution at 1 × 10^9^ CFUs per mouse for 24 h. Meanwhile, the mice solely treated with saline solution were as the no antibiotic control. While infected mice were treated with 0.5 µg mL^−1^ ciprofloxacin and 2 µg mL^−1^ tetracycline were served as antibiotic treatments. More experimental details could be found in the supporting information.

##### Ethics Statement

All animal protocols were approved by the Genentech Institutional Animal Care and Use Committee at the China Agricultural University (SYXK, 2016‐0008). The experimental procedures involving mice and rats were gained an approval (SCXK, 2016‐006).

##### Confocal Laser Scanning Microscopy Analysis

For static images, fixed and stained intestinal or cellular samples were captured by a Leica SP8 confocal microscope. 3D images were taken by capture all the *X*‐, *Y*‐, and *Z*‐axis sections. For analyzing the location of internalized bacteria, the *Z*‐axis section was cut every 1 or 2 µm. Images were analyzed and merged by the LAS AF Lite software (Leica).

##### Antimicrobial Activity Analysis

Extracellular minimal inhibitory concentrations (MICs) and minimal bactericidal concentrations (MBCs) were used to determine the antimicrobial activity of antibiotics in extracellular environment. The intracellular MBCs of antibiotics were used to define the antimicrobial activity of antibiotics for that internalized bacteria in cytoplasm, according to a previous publication^[^ ^33^ ^]^ with slight modifications. More details of the experimental protocols of antimicrobial activity used in this work were expanded in Supporting Information.

##### Virulence Factor Assays

Two kinds of bacterial toxins including non‐hemolytic enterotoxin of *B. cereus* (Nhe) and *α*‐toxin of *S. aureus* (AT), were tested. Nhe (23 ng mL^−1^), AT (50 ng mL^−1^, Sigma‐Aldrich), and their corresponding neutralizing antibodies (anti‐NheB mAb 1E11, 2 µg mL^−1^ and anti‐*α*‐toxin mAb, MEDI4893*, 5 µg mL^−1^, Sigma‐Aldrich) were involved as well. Ribbit anti‐IgG antibody (2 µg mL^−1^, Beyotime) was used as a negative control. IEC‐6 cells, bacteria (*B. cereus*, *S. aureus*, and *E. coli*), and sole bacterial toxin in the presence or absence of its antagonist were simultaneously added. After incubation for 2 h, the numbers of internalized bacteria were counted as previous description above. Lastly, the damage of phospholipid resulting in increased levels of choline were detected by a Phospholipid Assay Kit (Sigma‐Aldrich) and lactate dehydrogenase (LDH) in the media from damaged cells were determined by a LDH Release Kit (Beyotime), according to their instructions.

##### Autophagy Analysis

To track autophagy, IEC‐6 cells were seeded on glass coverslips (14 mm, NEST) in a 24‐well plate and transfected with modified adenoviruses (Ad‐mcherry‐GFP‐LC3B and Ad‐GFP‐p62, Beyotime) at 2 × 10^6^ plaque forming units (PFUs), according to the product's instruction. Then, labeled cells were cultured in DMEM with 10% FBS in the presence of *B. cereus* NVH0075/95 or *E. coli* ATCC29522, under the treatments of either ciprofloxacin or tetracycline (0.5 µg mL^−1^ ciprofloxacin and 4 µg mL^−1^ tetracycline for *B. cereus*; 0.25 µg mL^−1^ ciprofloxacin and 0.5 µg mL^−1^ tetracycline for *E. coli*) for 8 h. Lastly, coverslips were collected and fixed for confocal microscope analysis. LC3B was shown as green or red or the both, whereas the protein of p62 was shown in green.

##### Acidified Lysosome Tracking

IEC‐6 cells were incubated with LysoTracker Red DND‐99 (1 µm L^−1^, Invitrogen) for 35 min to stain lysosomes, then were incubated with Hoechst 33 342 (5 µg mL^−1^, Sigma‐Aldrich) for another 5 min to stain nuclei. Lastly, the fluorescent images were captured by a SP8 confocal microscope (Leica). The numbers of internalized *B. cereus* that colocalized in acidified compartments were counted by ImageJ software (National Institute of Mental Health). The fluorescence of acidified compartments dynamic curves (lysoTracker) were detected by a plate reader (Tecan Infinite 200pro) at the excitation wavelength of 520 nm and emission wavelength of 560 nm for every 1 h last for 24 h.

##### Western Blot

Western blot was utilized to analyze the expression of LC3 and p62/SQSTMI. Rapamycin (100 nm, abcam) and chloroquine (10 µm, Cell Signaling Technology) were used to induce and inhibit autophagy, respectively. The primary antibodies included rabbit anti‐LC3, rabbit anti‐p62/SQSTMI, rabbit anti‐LAMP1 (Abcam) and mouse anti‐*β*‐actin antibodies (Proteintech), secondary antibodies were goat anti‐rabbit and goat anti‐mouse antibodies (Beyotime). Gray values of protein bands were quantified by ImageJ software.

Related V‐ATPase complexes (V0 and V1 sectors) were also detected by Western blot. IEC‐6 cells were pre‐incubated with bafilomycin A1 (a specific inhibitor of V0‐sector, 100 nm) for 1 h. The lysosome‐related organelles were separated by a Lysosome Isolation Kit (Sigma‐Aldrich) used for Western blot. The primary antibodies included rabbit anti‐ATP6V0D, anti‐ATP6V1D (Abcam), anti‐Phospho‐mTOR (Ser2448) (Cell Signaling Technology), and mouse anti‐*β*‐actin antibodies (Proteintech), secondary antibodies were goat anti‐rabbit and goat anti‐mouse antibodies (Beyotime). Gray values of protein bands were quantified by ImageJ software.

##### ATP and ROS Detection

The ATP levels of infected IEC‐6 cells were detected by an Enhanced ATP Assay Kit (Beyotime) based on the manufacturer's instruction. Total ATP levels of IEC‐6 cells were quantified by firefly luciferase detection using a luminometer (Tecan Infinite 200pro) and calculated the ATP concentrations (nmol µg^−1^) were based on ATP standard curve. Intracellular ROS accumulations of IEC‐6 cells were detected by Reactive Oxygen Species Assay Kit (Beyotime) according to the instruments’ instruction. When ROS release from cells, the ROS probe, DCFH‐DA hydrolyzed to DCFH (green fluorescence), which fluorescence value (a.u.) was quantified by a plate reader (Tecan Infinite 200pro) at the excitation wavelength of 488 nm and emission wavelength of 520 nm.

##### Acid Phosphatase (ACP) Determination

IEC‐6 cells were infected with *B. cereus* at an MOI of 40 under 0.5 µg mL^−1^ ciprfloxacin treatment at 37 °C for 8 h. The activity of ACP (U mL^−1^) from cultural supernatants was detected by an Acid Phosphatase Assay Kit (Beyotime) though quantification of the consumption of phosphatase chromogenic substrates (Para‐nitrophenyl phosphate, pNPP), according to the manufacturer's instruction.

##### Mitochondrial Membrane Potential Detection

IEC‐6 cells were infected with *B. cereus* (MOI = 40), treated with 0.5 µg mL^−1^ ciprofloxacin at 37 °C for 8 h. NAC (5 mm) was used as a ROS scavenger. Then mitochondrial membrane potential (*Δψm*) of IEC‐6 cells was analyzed by a JC‐1 Kit (Beyotime). Cells with high *Δψm* showed in red and those with low *Δψm* in green fluorescence. The radio of JC‐1 green to red reflects the ability of JC‐1 aggregates into monomers, which represents the loss of *Δψm*. JC‐1 fluorescent images were captured by a SP8 confocal microscope (Leica).

##### Immunofluorescence

Cells were fixed with 4% (v/v) paraformaldehyde (Sigma‐Aldrich) and permeabilized with 0.3% (v/v) Triton X‐100 (Sigma‐Aldrich) for 3 min. Then cells were incubated with rabbit anti‐ATP6V0D1 primary antibody or rabbit anti‐LAMP1 antibody (a dilution of 1: 200, Abcam) at 4 °C overnight. Then cells were incubated with Cy3‐labeled goat anti‐rabbit IgG (H + L, a dilution of 1:500, Beyotime) at room temperature for 1 h. Nuclei were stained with DAPI for 2 min. The images of ATP6V0D1 were captured by a SP8 confocal microscope (Leica).

##### Transcriptome Analysis

IEC‐6 cells (1 × 10^9^ cell per well) were seeded into culture dishes (9 cm size, Corning) and infected with 4 × 10^10^ CFUs of *B. cereus* (NVH0075/95) or *E. coli* (ATCC25922) in a 5% atmosphere at 37 °C for 24 h. Blank control was treated with DMEM medium. Cells were treated with 0.5 µg mL^−1^ ciprofloxacin alone were served as a negative control. IEC‐6 cells were treated with a mixture of *B. cereus* and ciprofloxacin as antibiotic exposure group. After 24 h treatment, the extracellular medium and bacteria were cleared and washed with PBS. Then total RNA of cells was extracted by RNA extracted kit (TaKaRa) for high‐throughput transcriptomic (RNA‐Seq) by Annoroad company. Differential expression analysis between sample groups was using DESeq2. Only genes with log_2_ fold change ≥ 2 and the *p* value (FDR, false discovery rate, BH method) < 0.1 were used for further analysis. All the changed genes were selected to use in the heatmap. Additionally, the production of IL‐8 in cells was detected by a Rat IL‐8 ELISA kit (BD Biosciences), according to the protocol.

##### Statistical Analysis

Statistical analysis was performed using GraphPad Prism 8 software. Results were expressed as means ± SEM. *p*‐values were calculated using unpaired *t*‐test with Bonferroni correction between two groups or one‐way ANOVA among multiple groups. Statistical significance was determined as *ns p* > 0.05, ∗*p* < 0.05, ∗∗*p* < 0.01, ∗∗∗*p* < 0.001. All animals were used for analysis unless the mice died. All experiments were performed on no less than three biological replicates.

## Conflict of Interest

The authors declare no conflict of interest.

## Author Contributions

X.L. and F.L. contributed equally to this work. J.S. and K.Z. conceived the project. X.L., F.L., and K.Z. performed experiments. X.L., S.D., and K.Z. did data analysis. X.L., F.L., and K.Z. wrote the manuscript. All authors read and approved the manuscript.

## Supporting information

Supporting Information
