# Supplementary material for: Sublethal Levels of Antibiotics Promote Bacterial Persistence in Epithelial Cells
Source: Adv Sci (Weinh). 2020 Jul 27;7(18):1900840. doi: 10.1002/advs.201900840 (PMC7509632; doi:10.1002/advs.201900840)
Supplement: Supplementary file 1 — Supporting Information [file ADVS-7-1900840-s001.pdf]

Copyright WILEY-VCH Verlag GmbH & Co. KGaA, 69469 Weinheim, Germany, 2020.

ADVANCED SCIENCE
Open Access

Supporting Information

for *Adv. Sci.*, DOI: 10.1002/advs.201900840

Sublethal Levels of Antibiotics Promote Bacterial Persistence in Epithelial Cells

Xiaoye Liu, Fei Liu, Shuangyang Ding, Jianzhong Shen, and Kui Zhu**

Supporting Information

Sublethal levels of antibiotics promote bacterial persistence in epithelial cells

Xiaoye Liu, Fei Liu, Shuangyang Ding, Jianzhong Shen, Kui Zhu**

Dr. X. Liu, F. Liu, Prof. J. Z. Shen, Prof. K. Zhu
Beijing Advanced Innovation Center for Food Nutrition and Human Health, College of Veterinary Medicine, China Agricultural University, No.2 Yuanmingyuan West Road, Beijing 100193, China

Dr. X. Liu, Prof. S. Y. Ding, Prof. J. Z. Shen, Prof. K. Zhu
National Center for Veterinary Drug Safety Evaluation, College of Veterinary Medicine, China Agricultural University, Beijing 100193, China

Prof. S. Y. Ding, Prof. J. Z. Shen
Beijing Key Laboratory of Detection Technology for Animal-Derived Food Safety and Beijing Laboratory for Food Quality and Safety, China Agricultural University, Beijing, China
E-mail: zhuk@cau.edu.cn (K.Z.); sjz@cau.edu.cn (J.S.).

Table of contents:

1. Bacterial strains
2. Mammalian cells
3. Mammalian cell infections
4. Antimicrobial activity analysis:
5. Mouse infections
6. Flow cytometry
7. Bacterial invasion dynamics
8. Antibiotic distribution
9. Starvation response analysis
10. Spore detection assay
11. Supporting Tables and Figures

1. Bacterial strains

Four Gram-positive bacteria and four Gram-negative bacteria were used in this study. In addition, *B. cereus* NVH0075/95, which was referred to as wild type throughout this study, was transformed with plasmid pGFP4412 to create the strain of *B. cereus* pGFP4412. More details of the bacterial strains used in this study were provided in Table S1. Routinely, bacteria were cultured in LB medium or LB agar at 37 °C with shaking at 200 r.p.m, while *V. parahaemolyticus* was grown in LB supplemented with 3% NaCl.

2. Mammalian cells

Four epithelial cell lines, two immune cell lines and two kinds of primary cell lines, were employed in this study (Table S2). *Δfas* or *ΔASK1* cells were mutants of Vero cells constructed by CRISPR-Cas9 knockout assay, accroding to the previously published method.[1] The *Δfas* or *ΔASK1* mutants were ensured at least 80% positive cells by puromycin (0.1 μg/ml, Sigma) selection,[1] cultured in MEM (Gibco) supplemented with 10% FBS (Invitrogen). A549, HepG2 and IEC-6 cells were cultured in DMEM (Gibco) supplemented with 10% FBS (Invitrogen) and 1% (w/v) sodium pyruvate (Sigma-Aldrich) at 37 °C in a 5% CO_2 atmosphere. Vero cells were grown in MEM (Gibco) supplemented with 1% FBS and 1% (w/v) sodium pyruvate. Both RAW 264.7 cells and SP2/0 cells were cultured in RPMI-1640 (Gibco), supplemented with 10% FBS and 1% (w/v) sodium pyruvate. RIMVECs were stored and cultured according to the previously published procedures.[2] RIECs were isolated from 1-day SD rats. Briefly, the ileum of rats was extracted immediately and the homogenates of ileac tissues were obtained after digesting in collagenase type I (Invitrogen) for 15 min at 37 °C. The cell suspension was centrifuged at 1000 × g for 5 min. Cell pellets were seeded into 6-well plates and cultured in DMEM supplemented with 20% FBS and 1% (w/v) sodium pyruvate for 24 h. Lastly, RIECs were identified based on their morphology and subsequently validated by immunofluorescent stain based on the presence of cytokeratin 18, using a rabbit-anti cytokeratin 18 antibody (CK18, Abcam).

3. Mammalian cell infections

Mammalian cells were seeded at 1×10^5 cells per well onto glass coverslips (14 mm, NEST) in 24-well culture plates (Corning) to form monolayers. Then bacterial colonies were scraped, and resuspended in PBS (0.01 M, pH=7.2, Gibco) to pre-incubated with pHrodo (Molecular Probes), except *B. cereus* pGFP 4412. Finally, 4×10^6 CFUs of bacteria were cocultured with mammalian cells. After co-culture for 2 h, antibiotics was used to inhibit extracellular bacteria. All the cells were collected for the following tests, and specific treatments will be indicated thereof. Generally, coverslips were washed thrice by PBS and fixed with 4% (v/v) paraformaldehyde for 10 min at

room temperature. F-actin of mammalian cells were stained using rhodamine phalloidin, while cellular nuclei by DAPI for confocal microscopy observation. Generally, overslips were washed thrice by PBS and fixed with 4% (v/v) paraformaldehyde for 10 min at room temperature. F-actin of mammalian cells were stained using rhodamine phalloidin, while cellular nuclei by DAPI for a Leica SP8 confocal microscopy observation.

4. Antimicrobial activity analysis

Extracellular minimal inhibitory concentrations (MICs) and minimal bactericidal concentrations (MBCs) were used to determine the antimicrobial activity of antibiotics in the extracellular enviroment. Briefly, serial two-fold dilutions of the antibiotics (ampicillin, ciprofloxacin, erythromycin, polymyxin B, tetracycline, rifampin and vancomycin, final concentrations range from 0.125 to 256 μg mL^{-1}, Sigma-Aldirch) in complete DMEM were tested in 96-well plates in quadruplicate, according to the microdilution method of Clinical and Laboratory Standards Institute (CLSI). Then, 1×10^6 CFUs of the tested bacteria were added 100 μl per wells in 96-well plates and cultured in the presence of antibiotics for 18-24 h at 37 °C in a 5% CO_2 atmosphere. Lastly, the extracellular MIC of each antibiotic was determined based on the dose, at which more than 90% bacterial growth is inhibited. And the extracellular MBCs were the minimum antibiotic doses that prevented the survival of bacteria with >99.9% bacteria dead on LB agar, which was detected based on the values of extracellular MICs.

The intracellular MBCs of antibiotics were used to define the antimicrobial activity of antibiotics for that interalized bacteria in cytoplasm, according to a previous publication [33] with slight modifications. Briefly, cells (RIECs, RIMVECs, IEC-6, A549, HepG2, Vero, RAW 264.7 and SP2/0) were seeded in a 96-well plate at a density of 1×10^5 cells per well and infected with *B. cereus* NVH0075/95 (MOI = 40) at 37 °C in a 5% CO_2 atmosphere, according to the procedures of mammalian cell infections. Extracellular bacteria were removed by gentamycin (100 μg mL^{-1}) incubated for 15 min and washed with PBS twice. The survival of internalized bacteria was assessed after the addition of tested antibiotics including ampicillin, ciprofloxacin, erythromycin, polymyxin B, tetracycline, rifampin and vancomycin (with final concentrations ranged from 20 μg mL^{-1} to 600 μg mL^{-1}) into plates for 24 h. Subsequently, cells lysed by DMEM supplemented with 0.1% BSA and 0.1% Triton X-100 and serial dilutions of the lysates were plated on LB agar for CFU counting.

5. Mouse infections

5-week-old female ICR mice ($n \geq 5$) were infected intragastrically with 200 μL bacteria (*B. cereus* NVH0075/95 and *E. coli* ATCC25922) in 0.9% saline solution at 1×10^9 colony-forming units

(CFUs) per mouse for 24 h. Meanwhile, the mice solely treated with saline solution were as controls. then, infected mice were treated with 0.5 μg mL^{-1} ciprofloxacin and 2 μg mL^{-1} tetracycline.

Small intestines including duodenum, jejunum and ileum, were collected to prepare for hematoxylin and eosin stain (HE staining). To localize bacteria in the tissues, bacteria were previously incubated with pHrodo (Molecular Probes) for 1 h prior to gavage with mice. The F-actin of intestinal sections and nuclei were stained with rhodamine phalloidin (Molecular Probes) and DAPI (Sigma-Aldrich) for Confocal Laser scanning microscopy analysis (Leica).

To analyze the antibiotic treatments effect on the exogenous *B. cereus* NVH0075/95 in small intestinal cells, *B. cereus* selective agar plates (Brilliance, OXOID) were employed to enumerate CFUs. Mice were treated as previous method above. The small intestines (including duodenum, jejunum and ileum) were extracted and washed with 100 μg mL^{-1} gentamicin for 1 h to remove extracellular bacteria. Then the intestines were homogenized and further lysed using DMEM supplemented with 0.1% bovine serum albumin (BSA) and 0.1% Triton X-100 (Sigma-Aldrich) to release internalized bacteria. Lastly, the suspension was collected to culture on selective agar plates at at 37 °C for 16 h.

6. Flow cytometry analysis

To record the proportion of infected cells and the survived status of internalizated *B. cereus*, flow cytometry method was employed according to a previous protocol.[4] First, *B. cereus* pGFP 4412 (green) were pre-incubated with pHrodo (red) for 1 h at 37 °C. Subsequently, all bacteria were washed thrice in PBS before infected IEC-6 cells at different MOIs ranged from 0.0125 to 100. Cells at various time-points (0, 2, 4, 6, 8, 12 and 24h) were washed with PBS twice and incubated with 100 μg mL^{-1} gentamicin for 15 mins to remove extracellular bacteria. Lastly, uninfected cells (no fluorescence), and cells infected with live bacteria (both green and red) and with dead bacteria (red only) were sorted by FACS. The proportion (at least 1×10^4 cells of each group) was analyzed by FACSDiva software (BD Biosciences).

In addition, IEC-6 cells were coclutured with pHrodo labeled *B. cereus* pGFP 4412 as mentioned above in the presence and absence of antibiotics (0.5 μg mL^{-1} ciprofloxacin, 0.25 μg mL^{-1} erythromycin, 4 μg mL^{-1} tetracycline, 0.625 μg mL^{-1} rifampin and 2 μg mL^{-1} vancomycin), at final concentrations of 0.5-fold of extracellular MICs for 24 h. Then, the proportion of infected cells was carried out using flow cytometry analysis. The gates of FACS assay was set by negative control, pHrodo color only and GFP color only. The cells had no bacteria showed no fluorescence, while cells infected with dead *B. cereus* (red fluorescence) or live *B. cereus* (both red and green

fluorescence or green fluorescence). Q1-1 represented bacteria were dead inside cells (red fluorescence), Q2-1 represented bacteria were live in the acid components (both red and green fluorescence), Q3-1 represented there was no bacteria inside cells (no fluorescence). Q4-1 represented bacteria were live and proliferation in cytoplasm (GFP fluorescence).

7. Bacterial invasion dynamics

Antibiotics (0.5 μg mL^{-1} ciprofloxacin, 0.25 μg mL^{-1} erythromycin, 4 μg mL^{-1} tetracycline, 0.625 μg mL^{-1} rifampin and 2 μg mL^{-1} vancomycin) at the final concentrations of 0.5-fold of extracellular MICs were simultaneously added with *B. cereus* NVH0075/95 labeled pHrodo at MOI of 40 in a 96-well plate, to coculture with IEC-6 cells for 8 h in complete DMEM. Then fluorescence of intracellular *B. cereus* NVH0075/95 was recorded by a plate reader (SpectraMax M5) with an interval of 20 min, at the excitation wavelength of 560 nm and emission wavelength of 585 nm.

8. Antibiotic distribution

IEC-6 cells were infected of *B. cereus* NVH0075/95 under antibiotic treatments in complete pentachromic DMEM (Gibico) for 24 h. Subsequently, the supernatants were centrifugalized at 10 000 g for 10 min, to remove cells and bacteria. Meanwhile, the monolayer cells were washed with PBS twice, collected and broken. Then the homogenates were further centrifugalized at 10 000 g for 10 min to obtain the supernatants. Lastly, all supernatants were filtered through 0.22 μm filter membrane (PALL) before liquid chromatography-mass spectrometry (LCMC-8045, Shimadzu) analysis. Total antibiotics were also detected by LC-MS/MS and used to calculated the lost antibiotics by total antibiotics minus the other detectable antibiotics. The lost antibiotics should include the antibiotics on cell surface, antibiotics lost during the sophisticated treatments before LC/MS measurements, consumed or degraded antibiotics by bacteria or epithelial cells, and antibiotics that bind to the components (e.g. protein) of the growth medium.

The supernatants in the glass vials were run though C18 column (2.1 × 100 mm, 3 μm, Shimadzu) for chromatographic separation at the flow rate of 0.30 mL/min. Mobile phase A was 0.1% formic acid in water and phase B was 0.1% formic acid in acetonitrile. The gradient elution program was: 0-1.0 min, 10% B; 1.0-2.0 min, 10-60% B; 2.0-3.0 min, 60-80% B; 3.0-4.0 min, 80-100% B; 4.0-5.5 min, 100% B; 5.5-5.6 min, 100-10% B; 5.6-7 min, 10% B. The injection volume was 3.0 μL. The parameters of mass spectrometry were: flow rate of sheath gas, 45; flow rate of auxiliary gas, 10; flow rate of cone gas, 0; electrospray voltage, 3.5 kV; temperature of ion tube, 320 °C; RF level of S-lens, 60; temperature of ion source is 350 °C. The concentrations of antibiotics in the supernatants were analyzed by MS in multiple- reaction monitoring (MRM)

mode under ESI positive ion mode (capillary voltage: 4.0 kV, desolvation temperature: 400 °C). Parameters for LCMC analysis were detailed in Table S4.

9. Starvation response analysis

The stavation response analysis of *B. cereus* and *E. coli* was used the qRT-PCR gene detection assay. Briefly, the RNA of bacteria from infected cell, EBSS (positive control) and DMEM (negative control) were collected and extracted to analyze the expression of *yjbM* and *yawC* in *B. cereus* and *reclA* and *sopT* in *E. coli*, respectively. The *16S rRNA* gene was served as reference gene. Primers were designed by Primer Designing Tool of NCBI online (https://www.ncbi.nlm.nih.gov/tools/primer-blast/) and showed on Table S3. Lastly, RT-PCR assay (SYBR Green qPCR Kit, TaKaRa) was performed based on previous methods[1] in triplicate by ABI quantstudio^{TM} 7 detection system (Applied Biosystem) and fold changes of gene expression were calculated by the $2^{-\Delta\Delta Ct}$ method.

10. Spore detection assay

IEC-6 cells were infected with *B. cereus* (MOI = 40) in the DMEM or treated with 0.5 μg mL^{-1} ciprofloxacin for 8 h. Both intracellular and extracellular bacteria were collected as previous method above. Then 1×10^8 CFUs *B. cereus* were collected to detect the spore genes including sporogenesis gene (*spo0A*) and spore germination gene (*Ger(x)C*). All the bacteria of different groups were used to determine the gene regulation by RT-PCR assay. The primers of spore genes were detailed in Table S3.

The numbers of spore were detected as following steps. Firstly, IEC-6 cells were infected with *B. cereus* at a MOI of 40 at 37 °C for 8 h with 0.5 μg mL^{-1} ciprofloxacin treatment. Then both of intracellular and extracellular bacteria were collected. Lastly, spores were dyed by a spore stain kit (Solarbio), according to the instruction and then counted by an inverted fluorescence microscope (Leica).

11. Supporting Tables and Figures

Table S1: Bacterial strains used in this study.

Strain	Description	Source / Reference
B. cereus NVH0075/95	Wild type, producing the tripartite components of Nhe toxin (A, B and C)	[1]
B. cereus pGFP4412	Transformant of *B. cereus* NVH0075/95, expressing GFP	This study
E. coli ATCC25922	Reference strain of Gram-negative bacteria for MIC test, producing heat-labile (LT) toxin	[6]
E. faecalis ATCC29212	Wide type (ST30), encoding virulence genes including collagen adhesion (*ace*) and hemolysin-cytolysin (*cyl* operon)	[7]
*K. pneumoniae*1202	Wild type (ST11), KPC-2 producer	[8]
P. aeruginosa PAO1	Wild type, producing pyocyanin	[9]
V. parahaemolyticus ATCC17802	Type strain, encoding virulence genes of Type III secretion systems	[5]
S. aureus ATCC29213	Reference strain of Gram-positive bacteria for MIC test, producing α-toxin	[10]
S. suis CQ2B50	A clinical isolate from piglet	This study

Table S2: Mammalian cells used in this work.

Cell type	Description	Source / Reference
A549	Lung carcinoma cell	ATCC CRM-CCL-185
HepG2	Human hepatocellular carcinoma	ATCC HB-8065
IEC-6	Rat small intestine cell	ATCC CRL-1592
RIEC	Rat intestine epithelial cell	This study
RIMVEC	Rat intestinal microvascular endothelial cell	[2]
RAW 264.7	Leukaemic monocyte/macrophage cell	ATCC TIB-71
SP2/0	Mouse hybridoma cell	IMM010.37.14
Vero	African green monkey kidney cell	ATCC CCL-81TM
Δfas	Knockout the *fas* gene in Vero cell	[1]
ΔASK1	Knockout the *ASK1* gene in Vero cell	[1]

Table S3: Sequences of primers used for quantitative RT-PCR.

**Primers**	**Sequences (5'to 3')**
relA-F	ACGTCTTTACGCCGAAAGGT
relA-R	TGGTTGTGACGTAACCGAGG
spoT-F	TGGGTTTTGAGGCGCTGTAT
spoT-R	TCGAGTGAAAACGCTGCTCT
yjbM-F	CCTGTGGCGAGCATACTTGA
yjbM-R	ACCGTCTGCAAAGGGTACAG
ywaC-F	AGGGCTGTGAGATTACGACG
ywaC-R	ACGTATTCCACACGGTTCGT
Spo0A-F	GACGAAAGTCTGACGGAGCA
Spo0A-R	TGCCACCTACGTATTACCGC
Ger(x)C-F	GGTTGCTCTGAGTTAGCGGA
Ger(x)C-R	GCCTTTTCCACCTTCTTGCG
16S rRNA-F	GAATCGCTAATCG
16S rRNA-R	GGGTTCCCCCATTCGGA

Table S4: Parameters for mass spectrometry analysis of antibiotics.

Compound	Precursor ions	Fragment ions	Collision energy (eV)
Ciprofloxacin	332	314.1*/288.2	21/19
Erythromycin	733.4	158.3*/576.3	31/21
Rifampin	823.4	791.45*/151.2	19/36
Tetracycline	445.25	410.15*/428.1	20/20
Vancomycin	725.4	100.1*/144.2	44/16

* quantitative ions

Table S5: Intracellular and extracellular MBCs (μg mL^{-1}).

Antibiotics	Targets	*E. coli*						*S. aureus*					*V. parahaemolyticus*				
		Intracellular MBCs					Extracellular MBCs	Intracellular MBCs				Extracellular MBCs	Intracellular MBCs (μg mL^{-1})				Extracellular MBCs
		RIECs	IEC-6	Vero	A549	HepG2		IEC-6	Vero	A549	HepG2		IEC-6	Vero	A549	HepG2	
**Ampicillin**	Cell wall	>600	400	>200	>600	600	8	600	>200	400	>600	2	>600	>600	400	>600	512
**Ciprofloxacin**	DNA gyrase	150	150	150	400	>600	0.125	200	200	200	600	0.25	150	150	200	150	0.5
**Erythromycin**	Ribosome	>600	200	>200	600	>600	256	400	200	400	400	4	200	200	400	200	8
**Polymyxin B**	Cell membrane	>600	100	70	200	200	1	>600	>200	400	>600	256	100	400	200	200	16
**Rifampin**	RNA polymerase	600	150	200	600	>600	32	200	200	400	200	0. 5	150	200	200	400	0.5
**Tetracycline**	Ribosome	400	>600	200	400	>600	1	150	200	400	400	2	150	150	600	150	32
**Vancomycin**	Cell wall	>600	>600	>200	>600	>600	256	>600	200	>600	>600	2	>600	>600	>600	600	256

Extracellular MBCs were the minimum antibiotic doses that prevented the survival of bacteria (with >99.9% bacteria dead), which was detected based on the extracellular MICs that prevented bacterial growth in DMEM. Intracellular MBCs were the minimum doses that prevented the survival of internalized bacteria in various mammalian cells (> denoted the continuous growth of bacteria under the maximum dose of antibiotics tested). Data are summarized from three different experiments.

Figures S1 to 10:

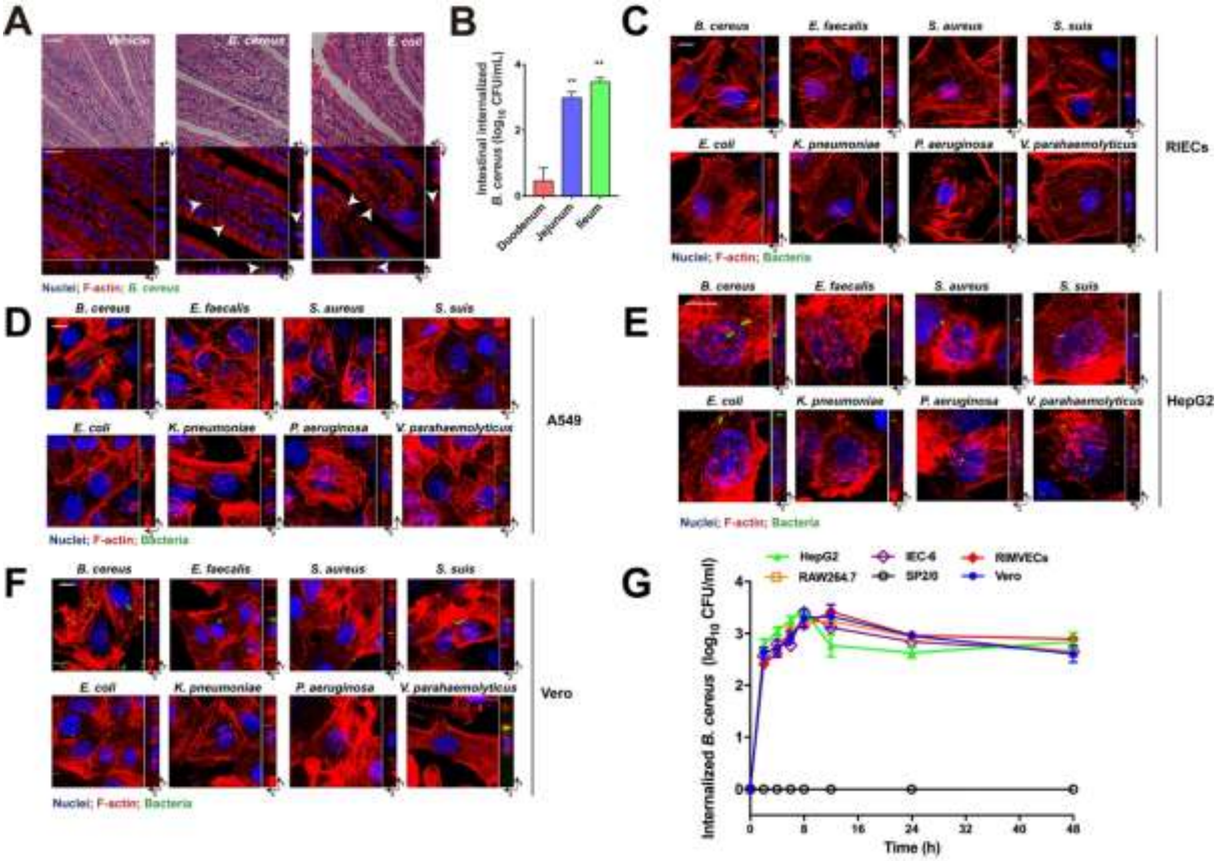

Figure S1: Bacterial interalizated in host intestine and different types of mammalian cells. (A) *B. cereus* and *E. coli* invaded into mice intestine. Mice were infected with a total of 1×10^9 CFUs *B. cereus* NVH0075/95 and *E. coli* ATCC25922 (pHrodo) for 24 h. *B. cereus* were shown in green (arrowheads). Sections of ileum were stained by HE (upper) and confocal images were visualized by rhodamine (bottom). Scale bars: 20 µm. **(B)** The numbers of *B. cereus* in the epithelial cells of duodenums, jejunums and ileums (n = 5 mice per group). Data are represented as mean ± SEM (** $P < 0.01$, n = 3). **(C-F)** Internalization of bacteria in rat primary intestinal epithelial cells (RIECs) (**C**), A549 (**D**), HepG2 **(E)** and Vero cells (**F**). Bacteria including *E. coli* ATCC25922, *E. faecalis* ATCC29212, *S. aureus* ATCC29213, *S. suis* CQ2B50, *K. pneumoniae* 1202, *P. aeruginosa* PAO1 and *V. parahaemolyticus* ATCC17892 were labeled by pHrodo (green) except *B. cereus* pGFP4412 expressing GFP (green). F-actin showed by rhodamine phalloidin (red) and nuclei visualized by DAPI (blue). Scale bars: 10 µm. **(G)** Growth dynamics of *B. cereus* NVH0075/95 in various cell lines. HepG2, IEC-6, RIMVECs, RAW264.7, SP2/0 and Vero cells were infected with *B. cereus* at different time points, and the numbers of intracellular surviving bacteria were determined by CFUs. Data represented at 3 independent repeats and as mean ± SEM.

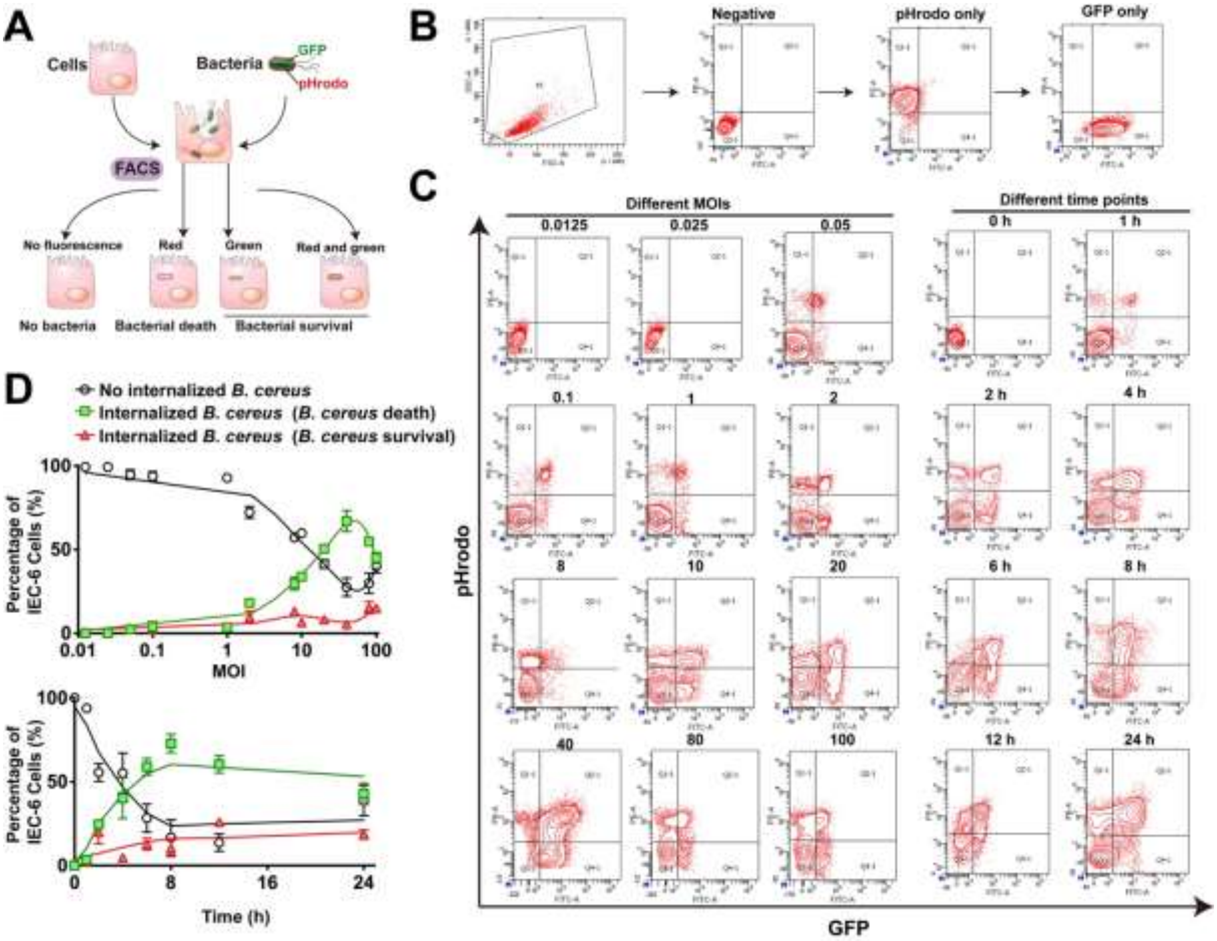

Figure S2: Growth curves of *B. cereus* in epithelial cells. (A) Scheme of flow cytometry assay. *B. cereus* pGFP4412 (green) were pre-incubated with pHrodo (red) and then infected IEC-6 cells. The cells with no bacteria showed no fluorescence, while IEC-6 cells with dead *B. cereus* shown red fluorescence, and with live *B. cereus* shown both red and green fluorescence or green fluorescence. **(B)** The gates of FACS assay was set by negative control, pHrodo color only and GFP color only. **(C)** Flow cytometry analysis of IEC-6 cells infected with *B. cereus* NVH0075/95 at different MOIs for 8 h and different time points at MOI of 40. Q1-1 represented *B. cereus* were dead inside cells (red fluorescence), Q2-1 represented *B. cereus* were live in the acid components (both red and green fluorescence), Q3-1 represented there was no *B. cereus* inside cells (no fluorescence). Q4-1 represented *B. cereus* were live and proliferative in cytoplasm (GFP fluorescence). **(D)** Proportion of cells that internalized *B. cereus* were calculated from **C** accroding to the different MOI (upper) or time (bottom) model.

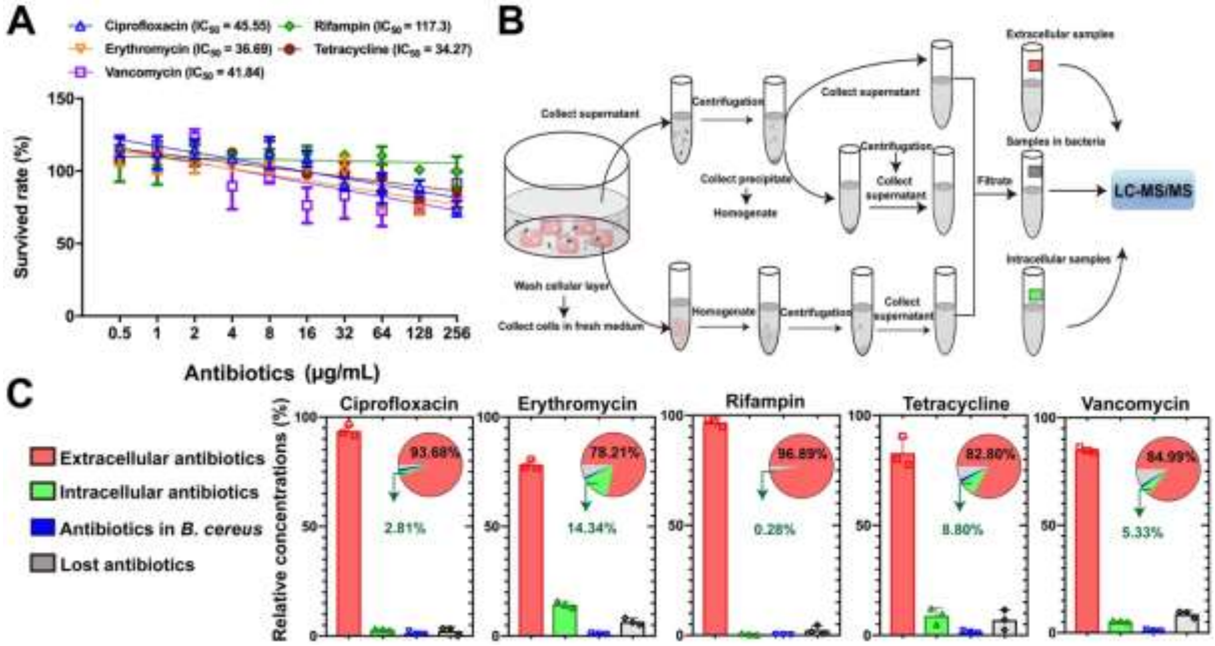

Figure S3: Antibiotics promoted bacterial invasion. (A) Effect of antibiotics on the survived rate of epithelial cells. IEC-6 cells were incubated with different concentrations of antibiotics (0 - 256 μg mL^{-1}) for 24 h. Cell proliferation and cytotoxicity of antibiotics was detected by WST-1 assay. **(B)** Scheme of antibiotic distribution detected by LC-MS/MS. **(C)** Distribution of antibiotics. The concentrations of extracellular antibiotics (red parts), intracellular antibiotics in the cytosol of IEC-6 cells (green parts), antibiotics in *B. cereus* (blue parts) quantified by LC-MS/MS. The loss antibiotics (gray parts) were calculated from the total antibiotics minus the other detectable antibiotics.

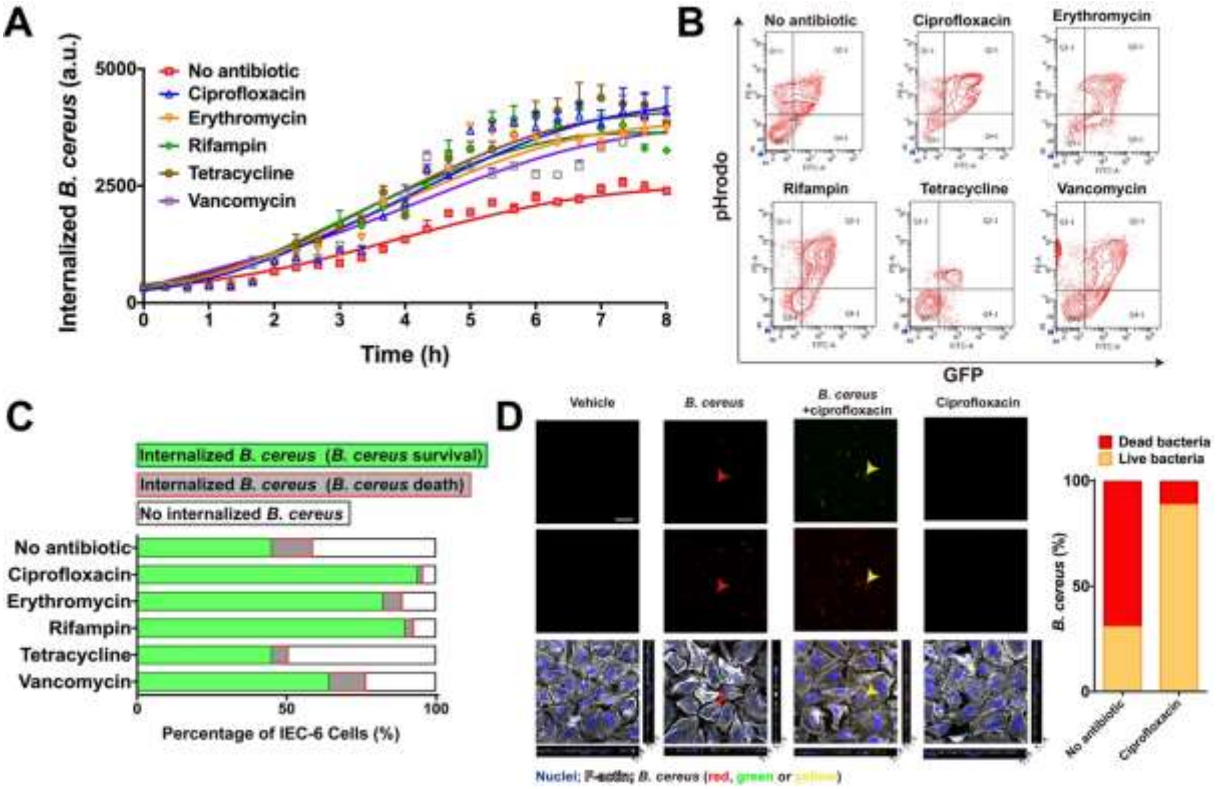

Figure S4: Sublethal levels of antibiotics promoted *B. cereus* survival in epithelial cells. **(A)** Dynamic curves of antibiotics enhanced invasion in a time dependent manner. IEC-6 cells and pHrodo pre-labeled *B. cereus* NVH0075 (MOI = 40) were measured in DMEM for 8 h. **(B, C)** Long-term exposure to antibiotics promoted *B. cereus* survival. IEC-6 cells infected with pHrodo pre-labeled *B. cereus* pGFP 4412 under antibiotics for 24 h. The populations of cells with live or dead *B. cereus* were analyzed by flow cytometry (at least 3×10^4 cells were utilized). **(D)** Ciprofloxacin facilitated *B. cereus* survival in epithelial cells. Images showed that the internalized *B. cereus* were pHrodo pre-labeled *B. cereus* pGFP 4412, which had both red and green fluorescence, while when *B. cereus* death, it would loss the green fluorescence. Red arrowheads represented bacterial death and yellow arrowheads showed bacterial alive. F-actin showed by rhodamine phalloidin (white) and nuclei visualized by DAPI (blue). Scale bars: 25 µm.

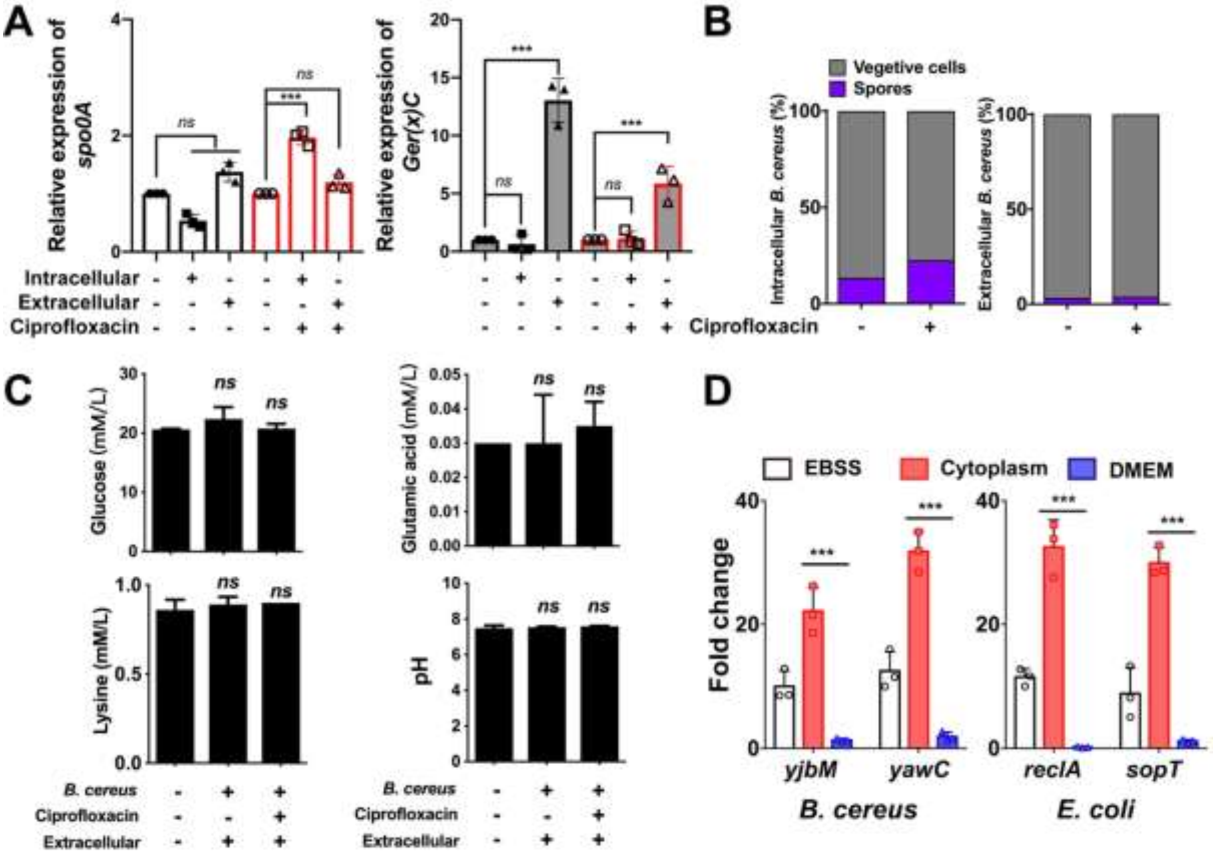

Figure S5: Sublethal levels of antibiotics promoted bacterial survival in an intracellular environment. (A) Spore genes of *B. cereus* were upregulated in the intracellular environment and enhanced under ciprofloxacin treatment. Sporogenesis gene (*spo0A*) and spore germination gene (*Ger(x)C*) were detected by RT-PCR in extracellular or intracellular environment. **(B)** The percentage of spores increased in intracellular environment under ciprofloxacin treatment. IEC-6 cells were infected with *B. cereus* under sublethal concentrations of ciprofloxacin treatment. All spores were counted by a spore stain kit and compared to the vegetable *B. cereus*. **(C)** Extracellular environment had no significant difference on glucose, glutamic acid, lysine and pH under 0.5 μ g mL^{-1}ciprofloxacin treatment. **(D)** Starvation responses of bacteria (*B. cereus* and *E. coli*) were activated in cells. The RNA of bacteria from either infected cell, EBSS (positive control) or DMEM (negative control) were collected and extracted to analyze the expression of *yjbM* and *yawC* in *B. cereus* and *reclA* and *sopT* in *E. coli*, respectively. Data are represented as mean ± SEM. ***$P <$ 0.001, n = 3 of independent experiments.

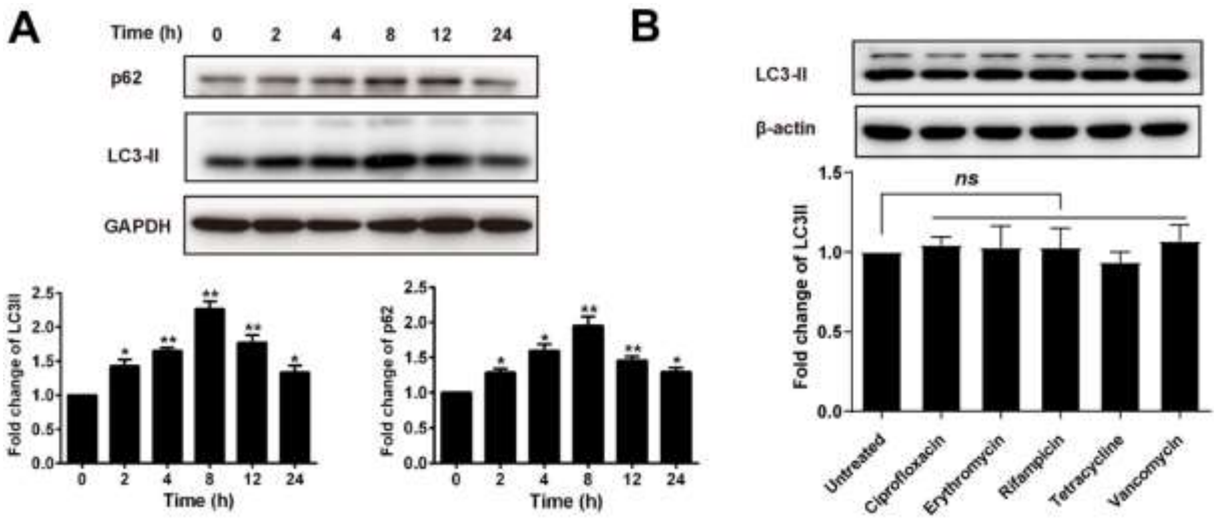

Figure S6: *B. cereus* interrupted autophagy. (A) *B. cereus* invasion induced autophagy arrest in a time dependent manner. IEC-6 cells were infected with *B. cereus* NVH0075/95 (MOI = 40) for different time points (0-24 h). Then the expression of LC3-II and p62 were analyzed by Western blot and normalized to the of levels GAPDH. **(B)** Antibiotics had no effects on the expression of lysosomal LC3-II. IEC-6 cells were treated with sublethal levels of antibiotics for 8 h, expression of LC3 -II was normalized to the levels of β-actin. Results are shown as means ± SEM (*$P < 0.05$; **$P < 0.01$; ***$P < 0.001$; *ns* $P > 0.5$, n = 3).

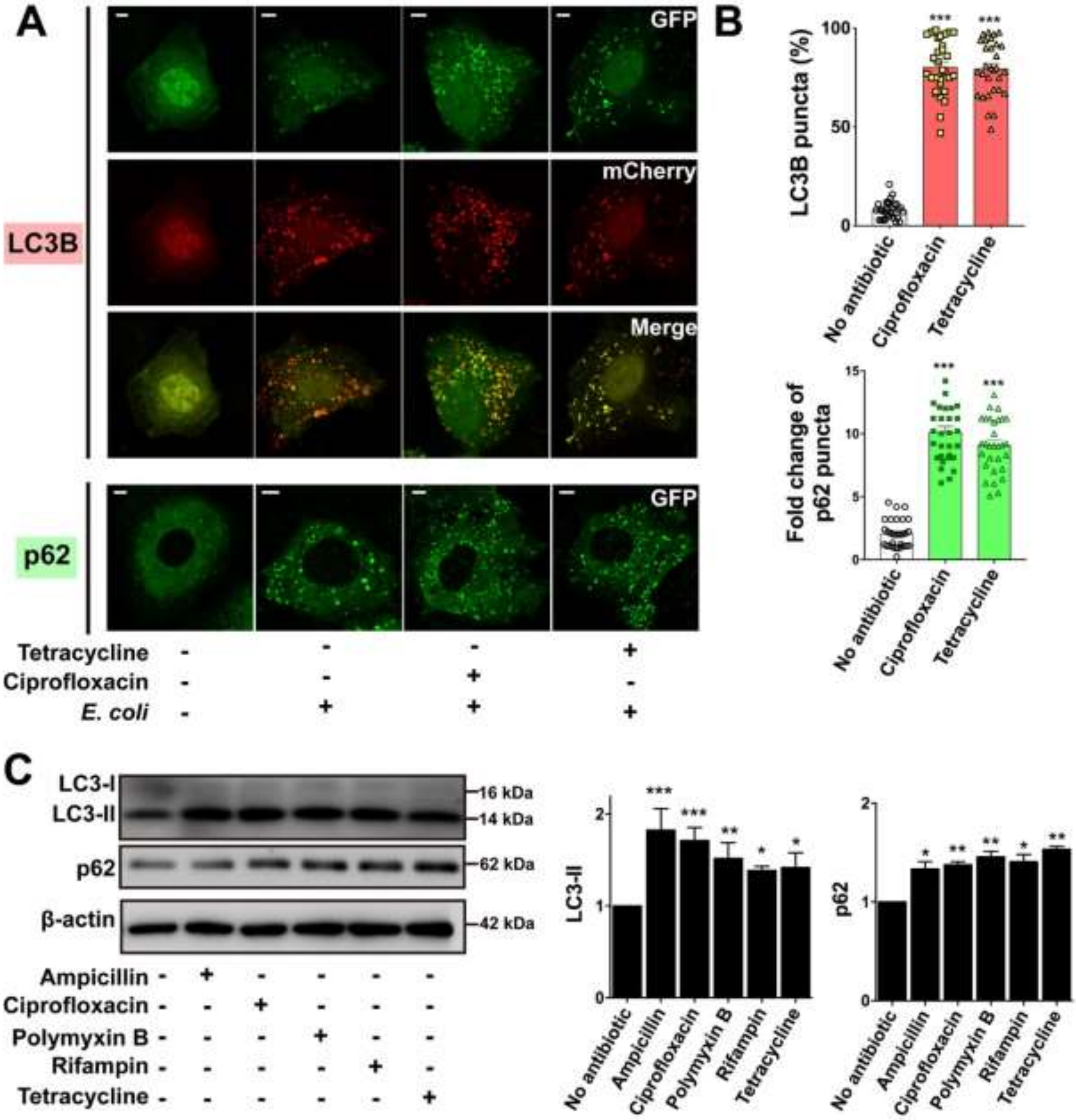

Figure S7: Sublethal levels of antibiotics interrupt autophagy to assist *E. coli* survival. **(A)** IEC-6 cells were transfected with modified adenoviruses (Ad-mcherry-GFP-LC3B and Ad-GFP-p62), then infected with *E. coli* ATCC25922 (MOI = 40) under antibiotic treatments (0.25 µg mL^{-1} ciprofloxacin and 0.5 µg mL^{-1} tetracycline) at concentrations of 0.5-fold extracellular MICs of *E. coli* for 8 h. Merge of LC3B presented either non-autophagy (diffused yellow LC3B), autophagy (red LC3B puncta) or autophagy arrest (yellow LC3B puncta). Scar bars = 3 µm. **(B)** Percentage of LC3B puncta and p62 puncta was quantified in (**A**). The percentage of LC3 was calculated from the ratio of yellow LC3B puncta to red LC3B puncta. The fold changes of p62 puncta was compared with untreated group. Both LC3B and p62 puncta were randomly selected for 30 cells (***$P < 0.001$) **(C)** Expression of

p62 and LC3 in IEC-6 cells infected with *E. coli* ATCC25922 (MOI = 40) with the treatment of antibiotics (Ampicillin, 4 μg mL^{-1}; ciprofloxacin, 0.25 μg mL^{-1}; polymyxin B, 0.5 μg mL^{-1}; rifampin, 8 μg mL^{-1}; tetracycline, 0.5 μg mL^{-1}) at concentrations of 0.5-fold extracellular MICs of *E. coli*. Protein extracts were analyzed by Western blot. Both LC3-II and p62 were normalized to the of levels β-actin. Data are shown as means ± SEM (*$P < 0.05$; **$P < 0.01$, ***$P < 0.001$, n = 3).

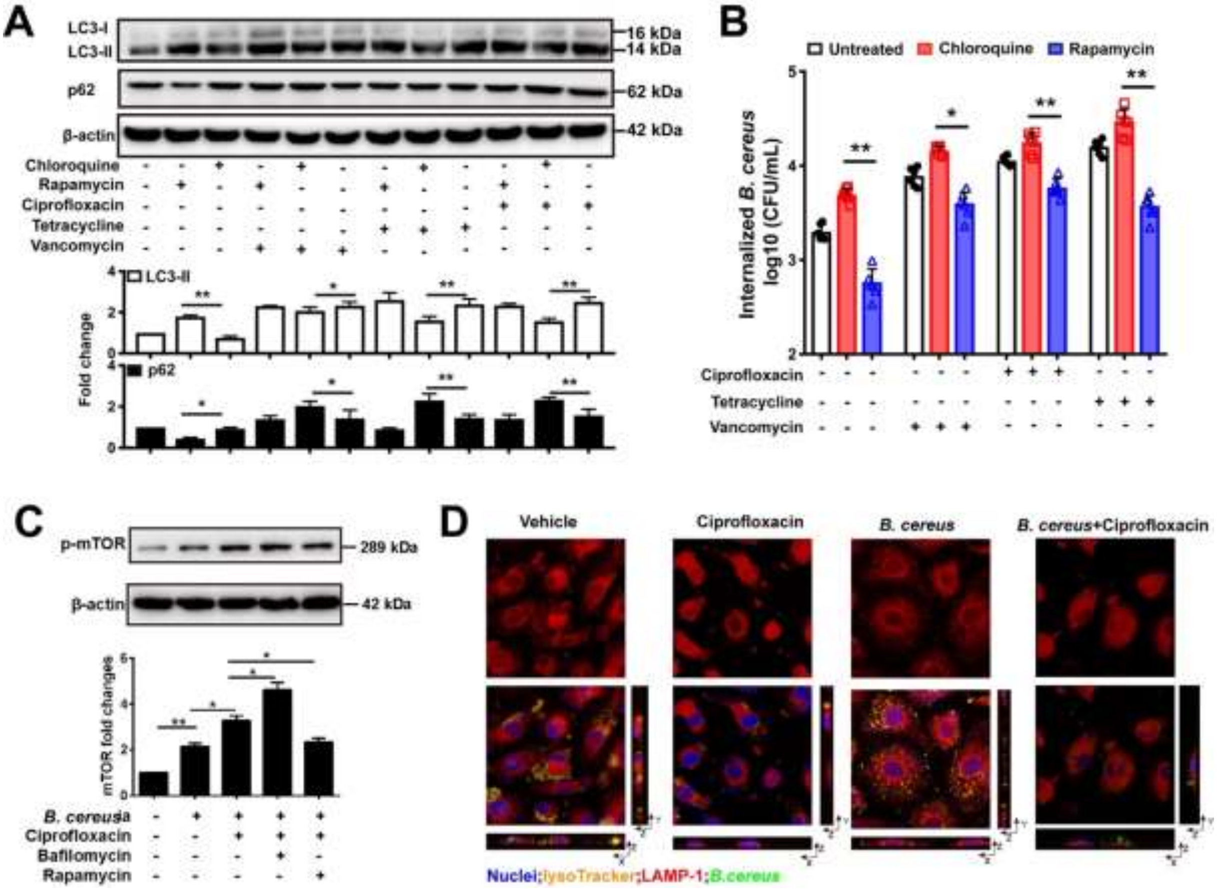

Figure S8: *B. cereus* interrupted autophagy could be enhanced by antibiotic treatments. **(A)** Autophagy inhibited by antibiotic treatments. IEC-6 cells were pre-incubated with rapamycin (an inducer of autophagy, 100 nM) and chloroquine (an inhibitor of autophagy, 10 μM) for 1 h. Then IEC-6 cells were infected with *B. cereus* NVH0075/95 (MOI = 40) under sublethal levels of antibiotic treatments (0.5 μg mL^{-1} ciprofloxacin, 0.25 μg mL^{-1} erythromycin, 4 μg mL^{-1} tetracycline, 0.625 μg mL^{-1} rifampin and 2 μg mL^{-1} vancomycin) . Expression of LC3-II and p62 were detected by Western blot. Both LC3-II and p62 were normalized to the levels of β-actin. **(B)** Antibiotics enhanced the survival of bacteria in cells due to autophagy arrest. The internalized *B. cereus* were detected by CFU counting. **(C)** Expression of p-mTOR in IEC-6 cells. **(D)** Ciprofloxacin lead *B. cereus* escaping from acid environment. Cellular lysosomes were marked with LAMP1 (red) and acid environments were tracked by lysotracker (yellow). Data are shown as means ± SEM (**P* < 0.05; ***P* < 0.01, n = 3).

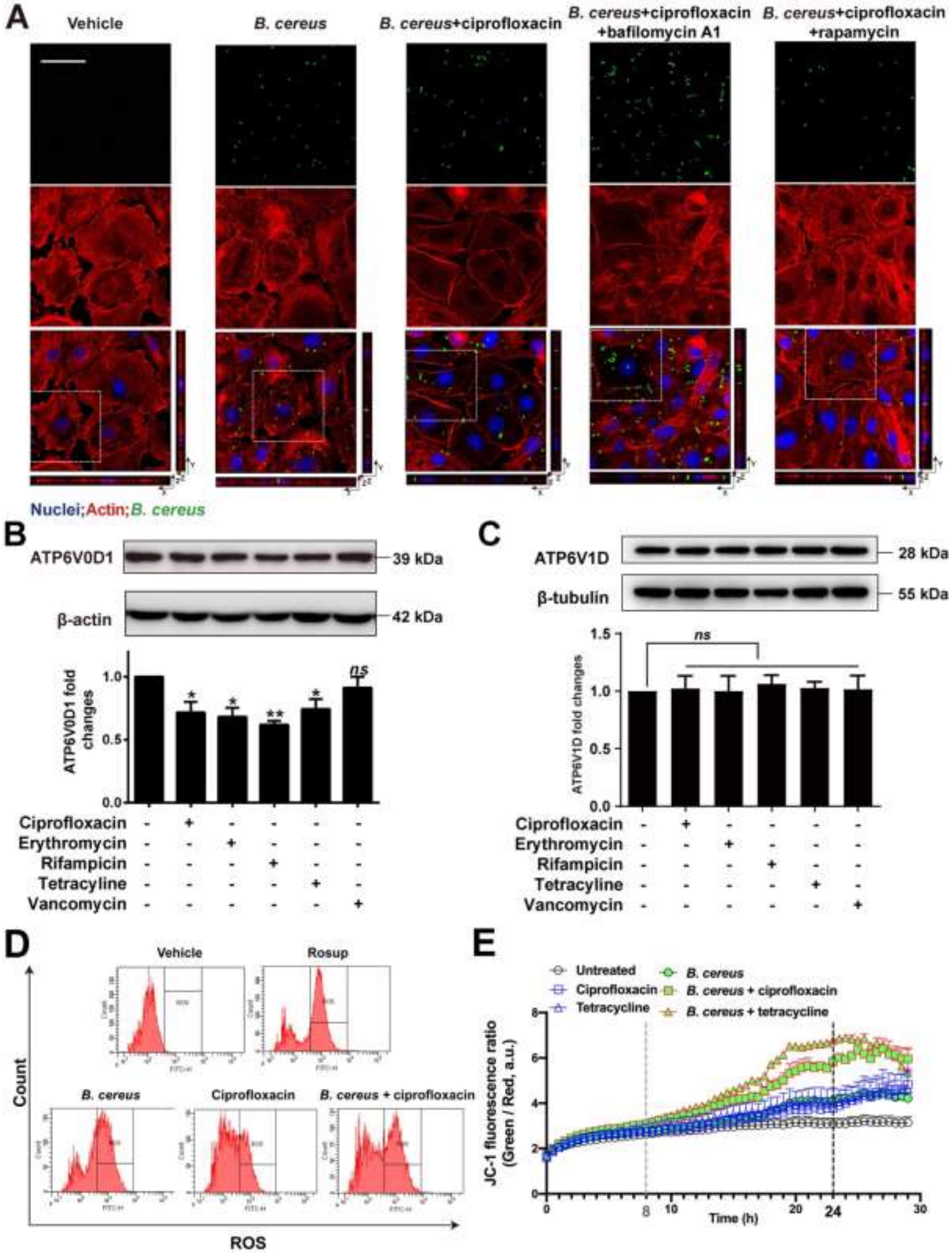

Figure S9: Antibiotics targeted mitochondria to lead ROS production to inhibit acidification. (A) Autophagy arrest promoted bacterial survival. IEC-6 cells were pre-incubated with bafilomycin A1 (an inhibitor of V-ATPase, 100 nM) and rapamycin (an inducer of autophagy, 100 nM) for 1 h. F-actin showed by rhodamine phalloidin (red) and nuclei visualized by DAPI (blue). Scale bar: 20 µm. **(B)** Expression of ATP6V0D1 in IEC-6

cells infected with *B. cereus* NVH0075/95 (MOI = 40) under antibiotic treatments. Expression of ATP6V0D1 was tested by Western blot assay. All proteins were normalized to the levels of β-actin. Data are shown as means ± SEM (compared to bacteria infected group, n = 3, **P* < 0.05, ***P* <0.001; *ns P* > 0.05. **(C)** Antibiotics had no effects on the expression of lysosomal ATP6V1D. IEC-6 cells were infected with *B. cereus* and treated with antibiotics at concentrations of 0.5-fold extracellular MICs for 8 h, expression of ATP6V1D were normalized to the levels of β-actin (n = 3, **P* < 0.05, ***P* <0.001; *ns P* > 0.05). **(D)** Antibiotics enhanced the accumulation of intracellular ROS. IEC-6 cells were treated with Rosup (125 μg mL^{-1}) as a positive control of ROS release and treated with DMEM as untreated control. Cells were tracked with DCFH-DC for 30 min. And intracellular ROS release were detected by Flow cytometry. **(E)** Antibiotics leaded mitochondrial membrane potential (MMP, ΔΨm) decrease. JC-1 was used to track the ΔΨm. Cells with high Δψm showed in red and those with low Δψm in green fluorescence. The radio of JC-1 green to red fluorescence reflecting the ability of JC-1 aggregates into monomers, which represented loss of Δψm. The curve showed the dynamic of JC-1 fluorescence from different time points (0-29 h).

References

[1] X. Liu, S. Ding, P. Shi, R. Dietrich, E. Martlbauer, K. Zhu, *Cell Microbiol.* **2017**, 19. doi: 10.1111/cmi.12684.

[2] X. Liu, H. Dong, M. Wang, Y. Gao, T. Zhang, G. Hu, H. Duan, X. Mu, *Immunol Res*. **2016**, 64, 133.

[3] S. M. Lehar, T. Pillow, M. Xu, L. Staben, K. K. Kajihara, R. Vandlen, L. DePalatis, H. Raab, W. L. Hazenbos, J. H. Morisaki, J. Kim, S. Park, M. Darwish, B. C. Lee, H. Hernandez, K. M. Loyet, P. Lupardus, R. Fong, D. Yan, C. Chalouni, E. Luis, Y. Khalfin, E. Plise, J. Cheong, J. P. Lyssikatos, M. Strandh, K. Koefoed, P. S. Andersen, J. A. Flygare, M. Wah Tan, E. J. Brown, S. Mariathasan, *Nature* **2015**, 527, 323.

[4] R. Avraham, N. Haseley, D. Brown, C. Penaranda, H. B. Jijon, J. J. Trombetta, R. Satija, A. K. Shalek, R. J. Xavier, A. Regev, D. T. Hung, *Cell* **2015**, 162, 1309.

[5] M. de Souza Santos, K. Orth, *mBio*. **2014**, 5, e01506.

[6] Y. Wang, G.-B. Tian, R. Zhang, Y. Shen, J. M. Tyrrell, X. Huang, H. Zhou, L. Lei, H.-Y. Li, Y. Doi, Y. Fang, H. Ren, L.-L. Zhong, Z. Shen, K.-J. Zeng, S. Wang, J.-H. Liu, C. Wu, T. R. Walsh, J. Shen, *Lancet Infect Dis.* **2017**, 17, 390.

[7] T. He, Y. Shen, S. Schwarz, J. Cai, Y. Lv, J. Li, A. T. Fessler, R. Zhang, C. Wu, J. Shen, Y. Wang, *J Antimicrob Chemother.* **2016**, 71, 1466.

[8] Y. Y. Liu, Y. Wang, T. R. Walsh, L. X. Yi, R. Zhang, J. Spencer, Y. Doi, G. Tian, B. Dong, X. Huang, L. F. Yu, D. Gu, H. Ren, X. Chen, L. Lv, D. He, H. Zhou, Z. Liang, J. H. Liu, J. Shen, *Lancet Infect Dis*. **2016**, 16, 161.

[9] Y. Liu, S. Ding, R. Dietrich, E. Martlbauer, K. Zhu, *Angew Chem Int Ed Engl*. **2017**, 56, 1486.

[10] S. Wendlandt, J. Shen, K. Kadlec, Y. Wang, B. Li, W. J. Zhang, A. T. Fessler, C. Wu, S. Schwarz, *Trends Microbiol*. **2015**, 23, 44.
